# The *Magnaporthe oryzae* effector Pwl2 alters HIPP43 localization to suppress host immunity

**DOI:** 10.1093/plcell/koaf116

**Published:** 2025-05-09

**Authors:** Vincent M Were, Xia Yan, Andrew J Foster, Jan Sklenar, Thorsten Langner, Amber Gentle, Neha Sahu, Adam Bentham, Rafał Zdrzałek, Lauren S Ryder, Davies K Kaimenyi, Diana Gómez De La Cruz, Yohan Petit-Houdenot, Alice Bisola Eseola, Matthew Smoker, Mark Jave Bautista, Weibin Ma, Jiorgos Kourelis, Dan Maclean, Mark J Banfield, Sophien Kamoun, Frank L H Menke, Matthew J Moscou, Nicholas J Talbot

**Affiliations:** The Sainsbury Laboratory, University of East Anglia, Norwich Research Park, Norwich NR4 7UH, UK; The Sainsbury Laboratory, University of East Anglia, Norwich Research Park, Norwich NR4 7UH, UK; The Sainsbury Laboratory, University of East Anglia, Norwich Research Park, Norwich NR4 7UH, UK; The Sainsbury Laboratory, University of East Anglia, Norwich Research Park, Norwich NR4 7UH, UK; Department of Molecular Biology, Max-Planck-Institute for Biology, Tübingen 72076, Germany; The Sainsbury Laboratory, University of East Anglia, Norwich Research Park, Norwich NR4 7UH, UK; The Sainsbury Laboratory, University of East Anglia, Norwich Research Park, Norwich NR4 7UH, UK; The Sainsbury Laboratory, University of East Anglia, Norwich Research Park, Norwich NR4 7UH, UK; Department of Biochemistry and Metabolism, John Innes Centre, Norwich Research Park, Norwich NR4 7UH, UK; The Sainsbury Laboratory, University of East Anglia, Norwich Research Park, Norwich NR4 7UH, UK; The Sainsbury Laboratory, University of East Anglia, Norwich Research Park, Norwich NR4 7UH, UK; The Sainsbury Laboratory, University of East Anglia, Norwich Research Park, Norwich NR4 7UH, UK; INRAE, UR BIOGER, Université Paris-Saclay, Palaiseau 91120, France; The Sainsbury Laboratory, University of East Anglia, Norwich Research Park, Norwich NR4 7UH, UK; The Sainsbury Laboratory, University of East Anglia, Norwich Research Park, Norwich NR4 7UH, UK; The Sainsbury Laboratory, University of East Anglia, Norwich Research Park, Norwich NR4 7UH, UK; The Sainsbury Laboratory, University of East Anglia, Norwich Research Park, Norwich NR4 7UH, UK; Department of Life Sciences, Faculty of Natural Sciences, Imperial College London, London SW7 2AZ, UK; The Sainsbury Laboratory, University of East Anglia, Norwich Research Park, Norwich NR4 7UH, UK; Department of Biochemistry and Metabolism, John Innes Centre, Norwich Research Park, Norwich NR4 7UH, UK; The Sainsbury Laboratory, University of East Anglia, Norwich Research Park, Norwich NR4 7UH, UK; The Sainsbury Laboratory, University of East Anglia, Norwich Research Park, Norwich NR4 7UH, UK; The Sainsbury Laboratory, University of East Anglia, Norwich Research Park, Norwich NR4 7UH, UK; USDA-ARS Cereal Disease Laboratory, University of Minnesota, 1551 Lindig Street, Saint Paul, MN 55108, USA; The Sainsbury Laboratory, University of East Anglia, Norwich Research Park, Norwich NR4 7UH, UK

## Abstract

The rice blast fungus *Magnaporthe oryzae* secretes a battery of effector proteins to facilitate host infection. Among these effectors, pathogenicity toward weeping lovegrass 2 (Pwl2) was originally identified as a host specificity determinant for the infection of weeping lovegrass (*Eragrostis curvula*) and is also recognized by the barley (*Hordeum vulgare*) Mla3 resistance protein. However, the biological activity of Pwl2 remains unknown. Here, we showed that the Pmk1 MAP kinase regulates *PWL2* expression during the cell-to-cell movement of *M. oryzae* at plasmodesmata-containing pit fields. Consistent with this finding, we provided evidence that Pwl2 binds to the barley heavy metal–binding isoprenylated protein HIPP43, which results in HIPP43 displacement from plasmodesmata. Transgenic barley lines overexpressing *PWL2* or HIPP43 exhibit attenuated immune responses and increased disease susceptibility. In contrast, a Pwl2^SNDEYWY^ variant that does not interact with HIPP43 fails to alter the plasmodesmata localization of HIPP43. Targeted deletion of 3 *PWL2* copies in *M. oryzae* resulted in a *Δpwl2* mutant showing gain of virulence toward weeping lovegrass and barley Mla3 lines, but reduced blast disease severity on susceptible host plants. Taken together, our results provide evidence that Pwl2 is a virulence factor that suppresses host immunity by perturbing the plasmodesmatal deployment of HIPP43.

## Teaser

The blast effector pathogenicity toward weeping lovegrass 2 acts as a virulence factor, which alters the cellular localization of host protein HIPP43 to suppress plant immunity.

## Introduction

Plant pathogens secrete virulence proteins called effectors during infection to suppress plant immunity and facilitate infection (Jones and Dangl 2006). Fungal pathogens, such as the devastating blast fungus *Magnaporthe oryzae*, utilize an extensive battery of more than 500 effectors (Wilson and Talbot 2009; Yan et al. 2023), but very few have been functionally characterized (Oliveira-Garcia et al. 2024). A subset of effectors are recognized by plant intracellular nucleotide-binding leucine-rich repeat (NLR) immune receptors to activate disease resistance. In rice for example, the paired NLRs Resistance Gene Analog 4 and 5 (RGA5/RGA4), Pyricularia-induced k-1 and k-2 (Pik-1/Pik-2), and Piks-1/Piks-2 confer resistance to *M. oryzae* strains that secrete Avirulence AVR1-CO39/AVR-Pia, AVR-Pik, or AVR-Mgk1 effectors, respectively (Ashikawa et al. 2008; Kanzaki et al. 2012; Cesari et al. 2013; Ortiz et al. 2017; Zhang et al. 2018; Sugihara et al. 2023). These effectors are members of the *Magnaporthe* Avrs and ToxB-like (MAX) family, which are sequence unrelated, structurally conserved (de Guillen et al. 2015), and overrepresented among effectors recognized by rice NLRs (Cesari et al. 2013; Maqbool et al. 2015). Pwl2 is a MAX effector first identified as a host specificity determinant that controls pathogenicity toward weeping lovegrass (*Eragrostis curvula*), a widely grown forage grass (Sweigard et al. 1995), and was recently shown to be recognized in barley by the Mla3 immune receptor (Brabham et al. 2024). While Pwl2 has been widely studied to understand effector secretion and delivery (Mosquera et al. 2009; Zhang and Xu 2014; Oliveira-Garcia et al. 2023), the biological function of Pwl2 is unknown.

Recent studies of MAX-effector perception by NLRs have implicated host small heavy metal–associated (sHMA) domain containing proteins as potential targets of some of these effectors, including Pwl2 (Zdrzałek et al. 2024). Small HMAs are highly expanded across plant species with putative functions including heavy metal detoxification and potential metallochaperones, but in most cases their functions are not known (Dykema et al. 1999; Suzuki et al. 2002; Gao et al. 2009). HMAs can be broadly classified into 2 families, heavy metal–associated plant proteins and heavy metal–associated isoprenylated plant proteins (HIPPs), which possess a C-terminal isoprenylation motif (CaaX, where “a” represents an aliphatic residue and “X” is any amino acid) important for membrane anchoring (Hála and Žárský 2019). The rice sHMA protein Pi21, for example, is a blast disease susceptibility factor (Fukuoka et al. 2009), which has led to deployment of loss-of-function alleles of *pi21*, as a recessive form of rice blast resistance (Fukuoka et al. 2009; Mutiga et al. 2021). Furthermore, differences in host sHMA protein repertoires are linked to host-specific, adaptive evolution of the APikL2 effector family (Bentham et al. 2021). Importantly, HMA protein domains have been identified as noncanonical immune sensory domains in some NLR proteins, such as the rice Pik-1 of the Pik-1/2 and RGA5 of the RGA5/4 pair receptors, respectively, essential for recognition of cognate *M*. *oryzae* effectors (Ashikawa et al. 2008; Kanzaki et al. 2012 ; Cesari et al. 2013 ; Maqbool et al. 2015). However, the function of sHMAs and their link to disease susceptibility is not understood, limiting our understanding of the role of MAX effectors in plant disease.

In this study, we set out to investigate the biological function of *PWL2*. We were motivated to understand how a broadly distributed effector such as Pwl2 functions during a susceptible interaction between *M. oryzae* and its host. We report that *PWL2* has undergone extensive duplication and expansion in copy number with many rice blast isolates possessing 3 to 5 copies, making its functional analysis challenging. Using clustered regularly interspaced short palindromic repeats (CRISPR)-associated with an RNA-guided endonuclease (Cas9), or CRISPR-Cas9 gene editing, however, we have generated a *Δpwl2* mutant confirming that Pwl2 is a host specificity determinant but also revealing a hitherto unrecognized virulence function. Furthermore, we demonstrate here that *PWL2* expression during infection is controlled by the Pmk1 MAP kinase, which regulates cell-to-cell movement by the fungus at plasmodesmata (PD)-containing pit fields (Sakulkoo et al. 2018). We reveal that Pwl2 targets the HIPP43 sHMA protein in barley, thereby displacing it from PD, and show that transgenic plants overexpressing either *PWL2* or HIPP43 have reduced immune responses and greater susceptibility to blast disease. Finally, we show that a Pwl2^SNDEYWY^ mutant unable to interact with HIPP43 fails to displace it from PDs and cannot complement the reduced virulence of *Δpwl2* mutants. When considered together, our study provides evidence that Pwl2 is a virulence factor in *M. oryzae* that suppresses host defense by relocalizing HIPP43 away from PDs to facilitate fungal invasion of plant tissue.

## Results

### Pwl2 is a cytoplasmic effector expressed during blast infection

**Figure 1.**
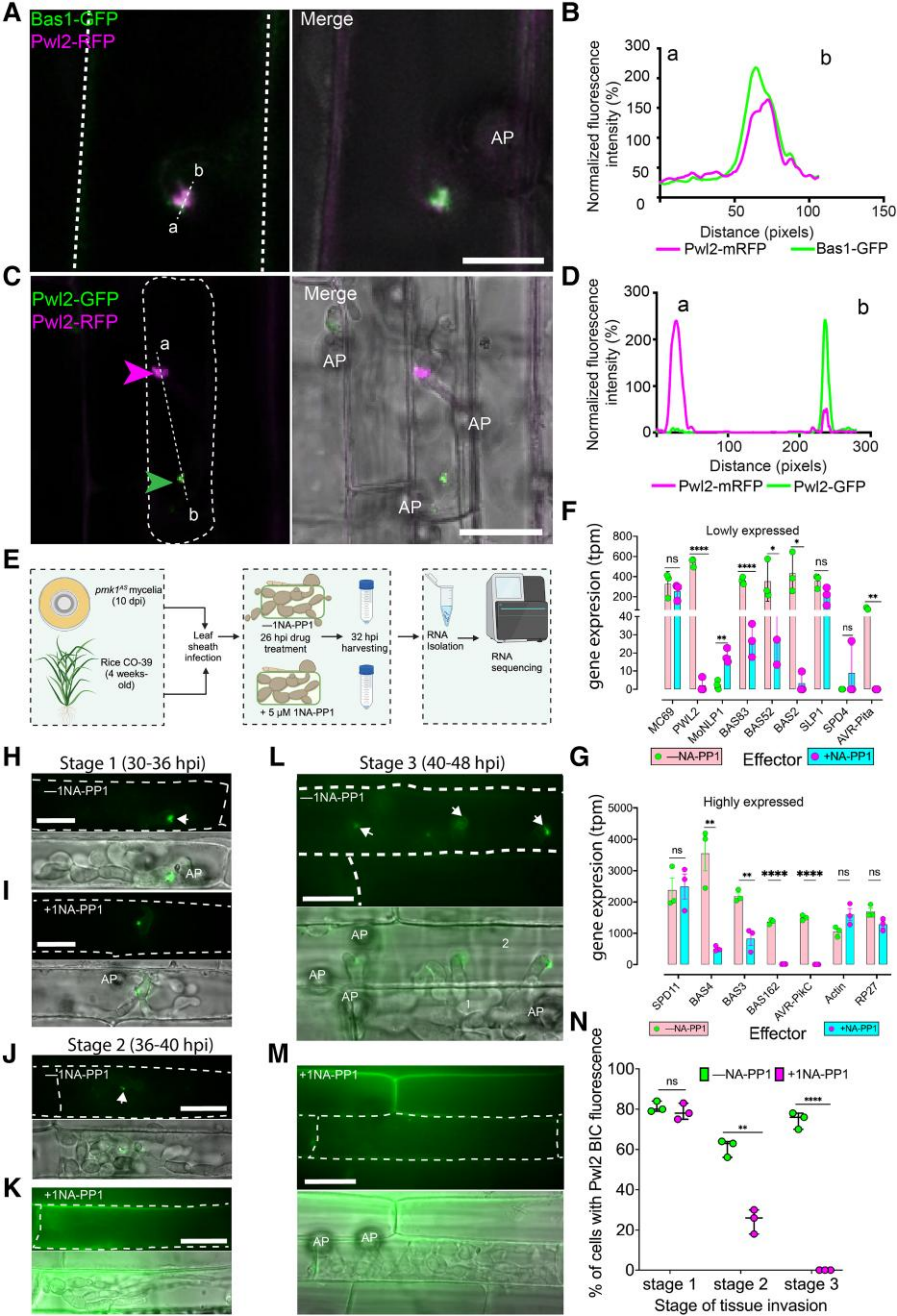
PWL2 expression is regulated in a Pmk1-dependent manner during host infection. **A** and **B)** Micrographs and line scan graph showing Pwl2 secreted through the BIC in rice cells during early infection. Conidial suspension at 1 × 10^5^ mL^−1^ of *M. oryzae* strain coexpressing 2 BIC-localized effectors; Bas1-GFP and Pwl2-mRFP were inoculated onto a susceptible cultivar Moukoto rice leaf sheath and images captured at 26 hpi. Fluorescence of the 2 effectors was observed as small punctate signals in the same BIC. **C** and **D)** Coinfection assay of rice leaf sheath with 2 different *M. oryzae* strains, one expressing Pwl2-mRFP and the other Pwl2-GFP at 30 hpi. Micrographs and line scan graph show there is an absence of mixed fluorescence confirming the BIC does not contain Pwl2 transferred from rice cells. BICs indicated by magenta arrowheads for mRFP and green arrowheads for GFP. **E)** Schematic illustration to describe the workflow used to test genes regulated in a Pmk1-dependent manner. Rice leaf sheaths were infected with a *M. oryzae pmk1^AS^* mutant spores before mock or 1NA-PP1 treatment. Treated and mock treated, infected leaf sheaths were trimmed and used for RNA isolation followed by sequencing. Figure created with BioRender https://biorender.com/. **F** and **G)** Bar charts to show that a subset of effectors is regulated in a manner that requires Pmk1. Gene expression is shown as transcripts per million at 32 hpi, **F)** showing lowly expressed effectors and **G)** highly expressed. Error bars represent SEM, and individual points represent 3 independent biological replicates. **H** to **M)** Micrographs showing expression of Pwl2-GFP by *M. oryzae pmk1^AS^* in leaf sheaths of a susceptible rice line CO39 using conidial suspension at 1 × 10^5^ mL^−1^. Fluorescence of Pwl2 at different stages of infectious hyphal progression, starting with early stage of infection, Stage 1 (30 to 36 hpi) where a newly differentiated bulbous hyphae is formed as per (Sakulkoo et al. 2018). Analysis was also carried out at later stages of infection including Stage 2 (36 to 40 hpi), where a primary invaded cell is filled with differentiated bulbous hyphae), and Stage 3 (40 to 48 hpi) where colonization of primary invaded cell is complete and there is full invasion of secondary invaded cells. Arrowheads indicate fluorescence in the BIC. The primary and secondary invaded cells are indicated with 1 and 2, respectively. Scale bars represent 20 *µ*m. To test Pmk1 inhibition, inoculated leaf sheaths were treated with 5 *µ*m 1NA-PP1 at 26 hpi. **N)** Inhibition of Pwl2 by 1NA-PP1 is quantified as percentage of cells showing Pwl2 fluorescence in the BIC (100 invaded cells were counted per replicate). Error bars represent SEM, and individual points represent independent biological replicate. For **F**, **G**, and **N)**, significance between groups of samples was performed using unpaired Student's *t*-test with Welch correction. ****P < 0.0001, ***P < 0.001, **P < 0.01, *P < 0.05; NS, no significant difference; AP, appressorium.

Expression of *PWL2* is specific to the initial biotrophic phase of plant infection, peaking at 48 h postinfection (hpi) (Yan et al. 2023) (Supplementary Fig. S1, A to C). The Pwl2 effector localizes in the biotrophic interfacial complex (BIC), a plant membrane-rich structure—that is clearly visible as a single bright punctum, initially at the tip of a penetration hypha and then adjacent to bulbous, branched invasive hyphae (Khang et al. 2010). The BIC is the predicted site of effector delivery (Kankanala et al. 2007; Giraldo et al. 2013; Oliveira-Garcia et al. 2023; Were and Talbot 2023), although experiments to date have not precluded that the BIC could be a site of effector sequestration from the host plant. To investigate this possibility, we generated a single *M. oryzae* strain expressing 2 BIC-localized effectors Pwl2-mRFP and Bas1-GFP and visualized their localization during infection of leaf sheath tissue in the susceptible rice cultivar Moukoto. The 2 effectors were observed as small punctate signals within the same BIC (Fig. 1, A and B). By contrast, when 2 different *M. oryzae* strains, Ina168 and Guy11, expressing Pwl2-GFP and Pwl2-mRFP, respectively, were used to simultaneously infect rice tissue, we observed that the BIC formed by each individual invasive hypha exclusively contained either Pwl2-GFP or Pwl2-mRFP, respectively, but never both fluorescence signals (Fig. 1, C and D). This is consistent with Pwl2 secretion by each fungal strain into the BIC, because sequestration of previously secreted Pwl2 from the plant cytoplasm to the BIC would result in a mixed GFP/mRFP fluorescence signal in the BIC. We conclude that Pwl2 is expressed early during infection and secreted to the BIC from where it is delivered into host cells.

### *PWL2* expression is regulated by the Pmk1 MAP kinase during invasive growth

The blast fungus invades rice tissue by means of pit field sites containing PD, which enable it to move between rice cells while maintaining integrity of the rice plasma membrane. PD conductance is also regulated by the fungus, enabling effectors like Pwl2 to be deployed in adjacent uninfected cells (Kankanala et al. 2007; Sakulkoo et al. 2018). Cell-to-cell movement by the fungus is regulated by the pathogenicity mitogen-activated protein kinase 1 (Pmk1 MAPK) pathway. An analog-sensitive mutant of Pmk1 has been shown to be unable to move through pit fields in the presence of the MAPK inhibitor 1NA-PP1 (Sakulkoo et al. 2018). Given that *PWL2* is expressed during the initial stages of infection, we decided to test whether it is regulated by Pmk1. We therefore reanalyzed RNA-seq data (Sakulkoo et al. 2018) (Fig. 1E) by separating raw reads of *M. oryzae* and *Oryza sativa* and quantifying transcript abundance using Kallisto, followed by determining differential expression using Sleuth. We found that *PWL2* is significantly downregulated in a *M. oryzae pmk1^AS^* mutant during cell-to-cell movement in the presence of 1NA-PP1 together with a subset of known effector genes—*BAS83*, *BAS52*, *BAS2, AVR-Pita, BAS3, BAS4, BAS162*, and *AVR-Pik-C* (Fig. 1, F and G). By contrast, effectors *MC69, SLP1, SPD4*, and *SPD11* did not show differential regulation, while *MoNLP1* showed upregulation (Fig. 1F). The expression of actin (MGG_03982) and the RP27 40S 27a ribosomal subunit genes (MGG_02872) were not significantly affected (Fig. 1G). We then used live-cell imaging to investigate Pwl2-GFP expression in a *pmk1^AS^* mutant during rice leaf sheath infection ±1Na-PP1. We initially observed Pwl2-GFP in the BIC at early stages of infection, unaffected (Stage 1) 30 to 36 hpi (4 to 10 h after 1NA-PP1 treatment) (Fig. 1, H and I), as previously reported (Sakulkoo et al. 2018), but by later stages of infection, Stage 2 (36 to 40 hpi, 10 to 14 h after 1NA-PP1 treatment) (Fig. 1, J and K), and Stage 3 (40 to 48 hpi, 14 to 22 h after 1NA-PP1 treatment) (Fig. 1, L and M), Pwl2-GFP fluorescence was significantly reduced (Fig. 1, K, M, and N). The reduction in Pwl2-GFP fluorescence was consistently associated with the stage at which *M. oryzae* traverses PD-containing pit field sites and enters adjacent plant cells. Taken together, we conclude that Pwl2 is regulated by the Pmk1 MAPK signaling pathway during cell-to-cell invasive growth by *M. oryzae.*

### *PWL2* is highly conserved in *M. oryzae*

**Figure 2.**
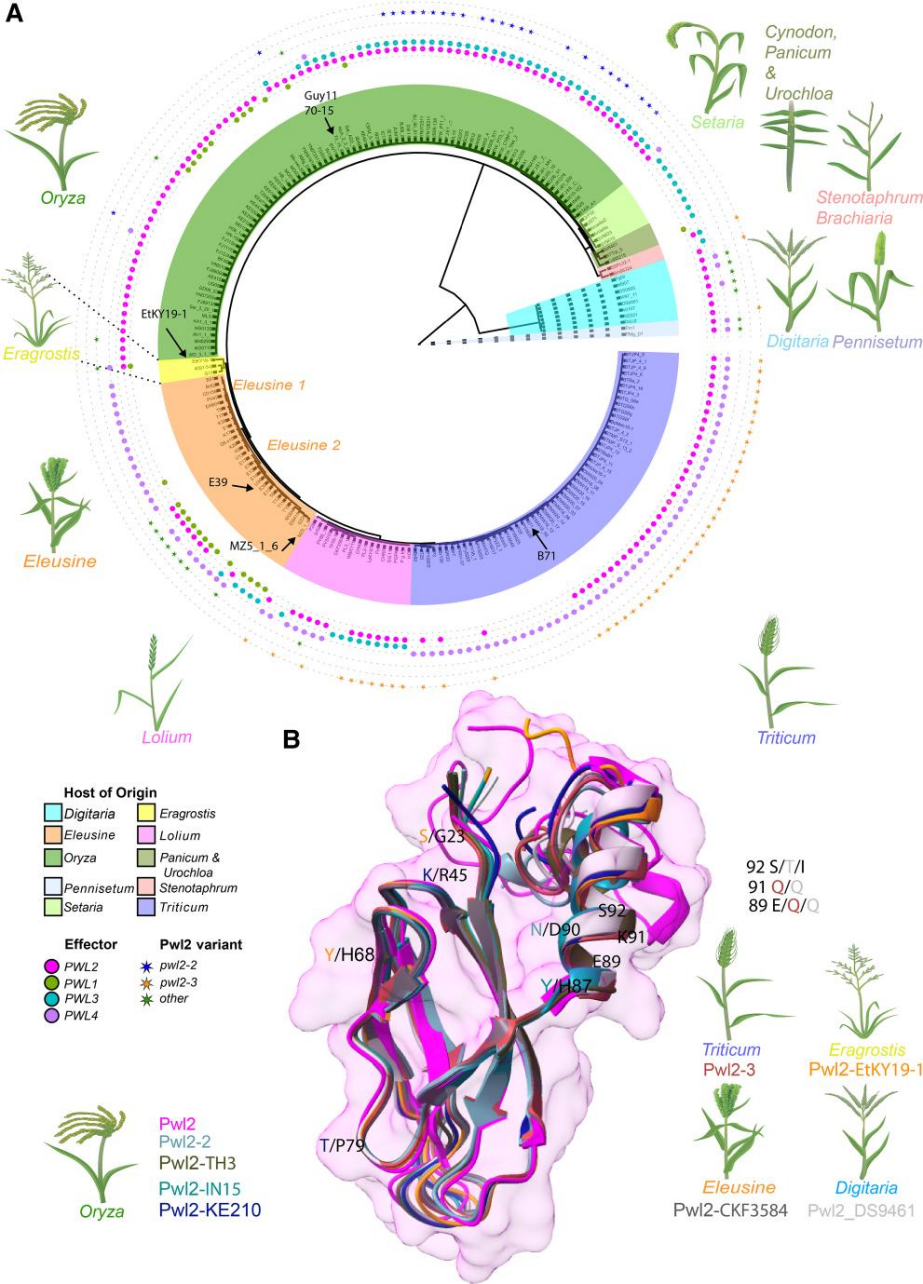
Pwl2 is highly conserved in isolates of *M. oryzae* and displays conserved structural features. **A)** Phylogenetic analysis and *PWL* gene family distribution in *M. oryzae*. A maximum parsimony tree (branch lengths are not drawn to scale) was generated using kSNP3 to include isolates from different host-limited forms of *M. oryzae* including isolates that infect *O. sativa* (rice), *Eleusine* spp. (finger millet), *H. vulgare* (barley), *Setaria* spp. (foxtail millet), *Triticum aestivum* (wheat), *Lolium* spp. (rye grass), *Brachiaria* spp. (armgrass millet), *Panicum* spp. (torpedo grass), *Eragrostis* spp. (weeping lovegrass), *Stenotaphrum* spp. (St. Augustine grass), *Cynodon* spp. (Bermuda grass), and *Urochloa* spp. (signal grass) (Ou 1980; Talbot 2003; Cruz and Valent 2017; Inoue et al. 2017), as well as *Magnaporthe* species that infect *Digitaria sanguinalis* (crabgrass) and *Pennisetum* spp. (pearl millet). We used Pwl1 (BAH22184.1), Pwl2 (QNS36448.1), Pwl3 (AAA80240.1), and Pwl4 (AAA80241.1) protein sequences to query the presence or absence of each gene using tblastn. The heatmap indicates the presence/absence of genes in the *PWL* family. *PWL1* is predominantly present in group EC-1I (Asuke et al. 2020) of *Eleusine*-infecting isolates and some *Oryza*-infecting isolates, but largely absent from other host-specific lineages, except one *Eragrostis*-infecting isolate, EtK19-1, and one *Cynodon*-infecting isolate, Cd88215, but not in *Digitaria* and *Pennisetum* spp. lineages. *PWL3* is present in most *Oryza*-infecting isolates, some *Setaria-*, *Brachiaria-, Stenotaphrum-,* some *Lolium-*, and some *Eleusine*-infecting isolates but largely absent in *Eragrostis* and *Triticum* lineages, as well as in *Digitaria* and *Pennisetum* lineages. *PWL4* is present in most *Eleusine-*, *Eragrostis-, most Lolium*-, most *Triticum*-, *Pennisetum*-, *Digitaria-*infecting isolates, but in only in 3 *Oryza*-infecting isolates. *PWL4* is however missing in *Setaria-, Brachiaria-* and *Stenotaphrum-*infecting isolates*. PWL2* is found in most host-limited forms of *M. oryzae* and related *Magnaporthe* species but absent in *Brachiaria, Setaria, Panicum, Cynodon*, and *Urochloa*. **B)** Superimposition of different Pwl2 variants predicted using AlphaFold3 onto the resolved Pwl2 structure (magenta) indicating region of polymorphism. Different colors represent different variants as follows: Pwl2-2 (teal), Pwl2-3 (brick red), Pwl2-TH3 (dark olive green), Pwl2-IN15 (dark teal), Pwl2-KE210 (blue), Pwl2-CKF3584 (dark gray), Pwl2-EtKY19-1 (orange), and Pwl2-DS9461 (light gray). The superimposition shows overall structural conservation in MAX-fold, without the signal peptide and the C-terminus. The variants of Pwl2 are named with corresponding isolate name and grouped according to host species. Varying residues are colored according to the genome from which they were identified.

*PWL2* belongs to a large gene family (Kang et al. 1995; Sweigard et al. 1995), but its conservation in the global rice blast population is not known. We therefore investigated *PWL* gene family distribution in isolates that infect a variety of different grass species (Fig. 2A; Supplementary Data Set 1). This revealed that *PWL2* is found in the majority of host-limited forms of *M. oryzae* and related *Magnaporthe* species, except for *Setaria* and closely related *Panicum-, Cynodon*-, and *Urochloa*-infecting isolates (although these were less well represented than other isolates) as shown in Fig. 2A. By contrast, *PWL1* is present in a subset (Asuke et al. 2020) of *Eleusine*-infecting isolates and some *Oryza*-infecting isolates, but largely absent from other host-specific lineages, except one *Eragrostis*-infecting isolate (EtK19-1) and one *Cynodon*-infecting isolate (Cd88215) (Fig. 2A). Similarly, *PWL3* is present in most *Oryza*-infecting isolates, most *Setaria-*, *Brachiaria-, Stenotaphrum-,* some *Lolium*-, and some *Eleusine*-infecting isolates. It is however, largely absent in *Eragrostis, Triticum*, *Digitaria*, and *Pennisetum* lineages. *PWL4* is present in most *Eleusine-*, *Eragrostis-,* most *Lolium-*, most *Triticum*-, *Pennisetum*- and *Digitaria*-infecting isolates, but in only three *Oryza*-infecting isolates and is missing in *Setaria*-, *Brachiaria*- and *Stenotaphrum*-infecting isolates (Fig. 2A). We conclude that the *PWL* gene family (Kang et al. 1995) is broadly distributed among blast fungus isolates infecting numerous grasses, but that *PWL2* is widespread across rice-infecting isolates and the majority of host-limited forms of *M. oryzae.*

Having determined that *PWL2* is broadly distributed in *M. oryzae*, we next sought to determine its allelic variability. In addition to a loss of recognition allele *pwl2-2*, which contains a single Asp-90-Asn substitution (Sweigard et al. 1995), we identified 14 new alleles of *PWL2* (Supplementary Fig. S2A). Notably, most polymorphic residues occur between positions His87 and Ser92, suggesting these residues might contribute to Pwl2 recognition by a cognate resistance gene (Supplementary Fig. S2A). Interestingly, we could only identify 1 variant of *PWL1*, 5 variants of *PWL3*, and 1 variant of *PWL4*, despite these effector genes occurring in finger millet, rice, wheat, and ryegrass-infecting lineages, respectively (Fig. 2A; Supplementary S2, B to D). It is possible that sample size for some host-specific lineages may lead to an underestimate of allelic variability in *PWL1, PWL3*, and *PWL4,* but from this analysis *PWL2* appears to be highly polymorphic and conserved, compared with other members of the gene family (Supplementary Fig. S2E). We also tested the ability of a subset of *PWL2* alleles to be recognized by barley *Mla3*. In some cases, we found variant *PWL2* alleles occurred in a *M. oryzae* isolate carrying *PWL1* (such as U34 and E39) or isolates with multiple copies of *PWL2* (e.g. TH3), precluding functional analysis, and not all isolates were available for testing. As expected, Guy11 did not produce lesions on cv. Baronesse (+*Mla3*) but was able to infect cv. Nigrate (−*Mla3*) (Supplementary Fig. S3, A to C). However, JUM1 (*pwl2-2*), BTJP4-16 (*pwl2-3*), and BN0293 (variant *pwl2*) all caused blast disease on cv. Baronesse (+*Mla3*), confirming that they contain loss of recognition *pwl2* alleles (Supplementary Fig. S3, A to C). Pwl2 has recently been structurally described as a MAX effector, containing a core β-sandwich fold formed of 2 antiparallel β-sheets, as well as a single α-helix, C-terminal to the β-sandwich fold (Zdrzałek et al. 2024). We were interested to understand how these variable residues affect Pwl2 recognition. We therefore mapped a subset of the identified variants of Pwl2 to the effector crystal structure. We noted that all polymorphic residues in *pwl2* alleles, such as the Glu-89-Gln, Lys-91-Gln and Ser-92-Ile substitutions in *pwl2-3,* were present in the C-terminal α-helix, a distinct region from the core MAX-fold (Fig. 2B). We conclude that this interface is likely used by immune receptors present in resistant barley (Mla3) and weeping lovegrass to bind Pwl2 and is potentially stabilized by the MAX-fold.

### *PWL2* has undergone copy number expansion in *M. oryzae*

**Figure 3.**
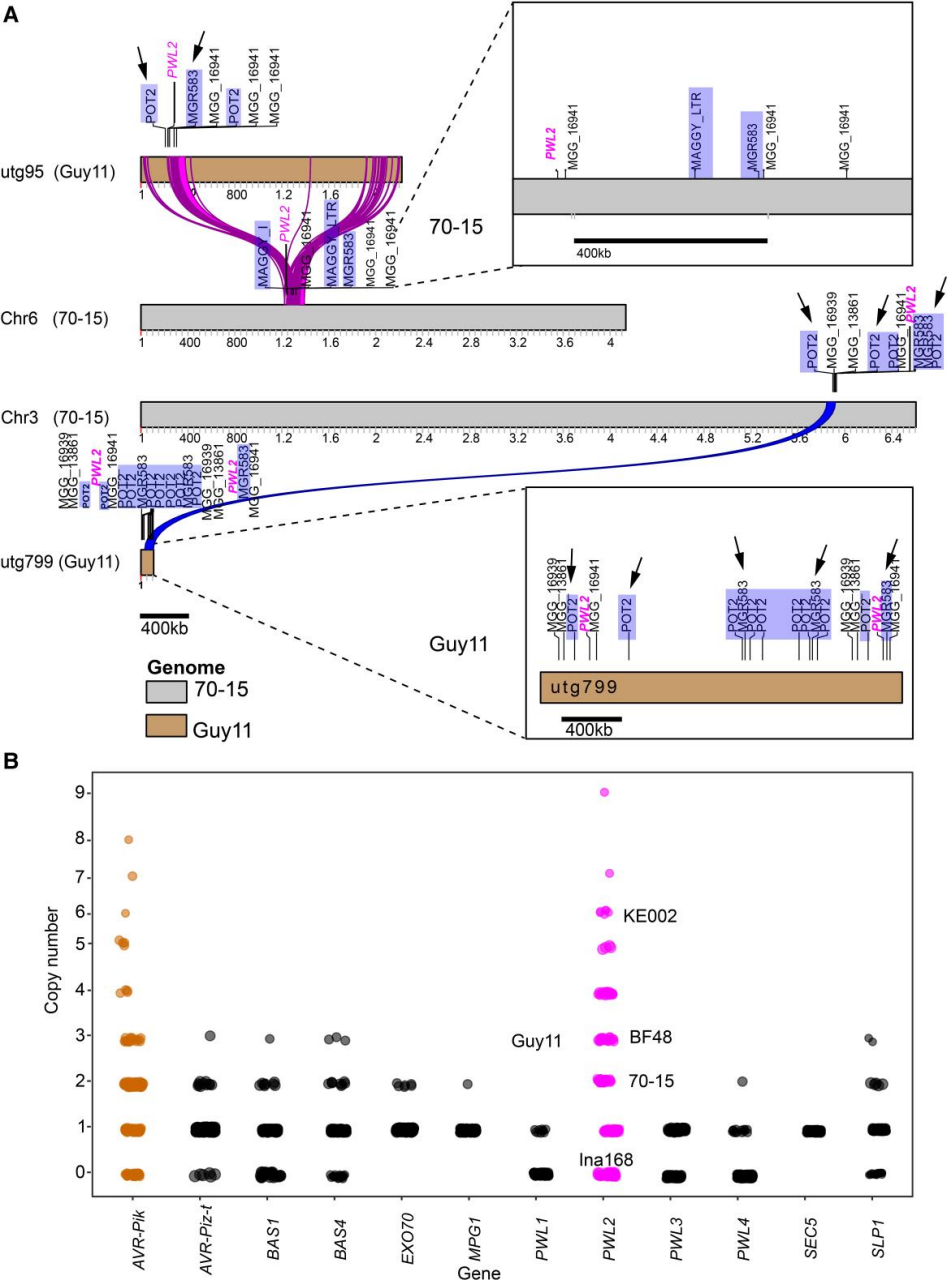
*PWL2* has undergone copy number expansion in *M. oryzae* field isolates. **A)** Schematic diagram showing the estimated chromosomal location of *PWL2* on multiple loci (Chr3 and 6) of the reference genome 70-15 and on equivalent region of assembled contigs of laboratory strain Guy11 genome. *PWL2* is flanked by *POT2* and *MGR583* repeated sequences suggesting a possible involvement in translocation of events into different loci in the genome. Arrows indicate location of *POT2* and *MGR58*, while *PWL2* is labeled in magenta. **B)** A k-mer analysis on sequenced raw reads was used to determine copy number variation of *PWL* family genes in different *M. oryzae* isolates. Plot shows high copy number of *PWL2* in analyzed genomes (*n* = 286) compared with the other gene family members, selected effectors *AVR-Piz-t, BAS1, BAS4*, and *SLP1,* and selected control genes *EX070, MPG1*, and *SEC5*. Similarly, *AVR-Pik* shows multiple copies in different isolates. Copy number of *PWL2* in selected isolates Guy11, KE002, BF48, 70-15 is indicated. Ina168 that lacks *PWL2* was used as a NC.

*PWL2* copy number appears to vary significantly among *M. oryzae* isolates. The *M. oryzae* reference genome assembly (strain 70-15) has 2 copies of *PWL2* on Chromosomes 3 and 6, annotated as MGG_13683 and MGG_04301, respectively (Dean et al. 2005). Southern blot hybridization (Supplementary Fig. S4, A and B), however, and analysis of long-read assembled genome sequence of *M. oryzae* Guy11 identified 3 copies of *PWL2* (Fig. 3A). *PWL2* loci were associated with *MGR583*, *POT2*, and *MAGGY* transposable elements, suggesting their potential involvement in genome rearrangements (Fig. 3A). To further assess *PWL2* copy number variation in *M. oryzae* isolates, we employed k-mer analysis to determine copy number variation of *PWL2* in 286 *M. oryzae* genomes (Supplementary Data Set 2). Copy number variation is common among effector-encoding genes, such as *AVR-Pik*, *AVR-Piz-t, BAS1, BAS4*, and *SLP1* but was particularly prevalent for *PWL2* with one isolate, for instance, containing 9 copies (Fig. 3B). We conclude that *PWL2* has expanded in copy number in *M. oryzae*.

### Pwl2 is both a host range determinant and a virulence factor

**Figure 4.**
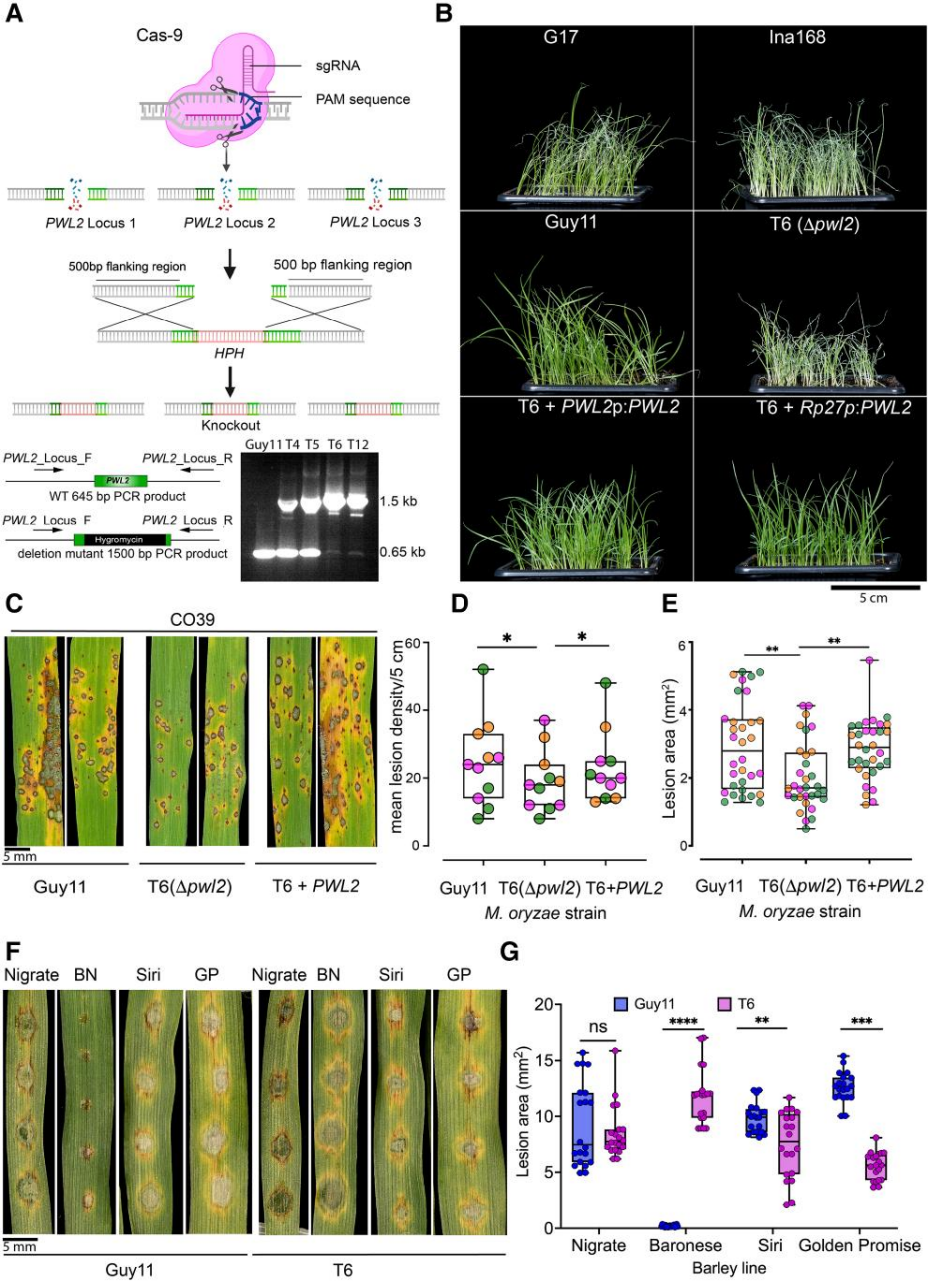
CRISPR-Cas9 *Δpwl2* mutants demonstrate Pwl2 as both host range and a virulence factor. **A)** Schematic illustration of the CRISPR-Cas9–mediated gene-editing process for inserting the *HPH* gene cassette at the *PWL2* locus. The guided sequence (sgPWL2) directs Cas9 to introduce a double-stranded break at the *PWL2* locus, with the DNA repair template containing the hygromycin resistance gene cassette and flanking regions of the *PWL2* gene. This leads to mutations in the form of indels or gene replacements. Figure created with BioRender https://biorender.com/. Positive mutants were identified by amplifying the hygromycin cassette from transformants with the 3 copies deleted. **B)** Comparison of disease symptoms in weeping lovegrass (*E. curvula*) infected with different *M. oryzae* isolates. Top panel shows typical disease lesions produced by *E. curvula*-infecting isolate G17 (−*PWL2*) and a rice-infecting isolate Ina168 (−*PWL2*). In the middle panel, CRISPR-Cas9 deletion mutants exhibit gain of virulence on weeping lovegrass compared with Guy11. In the bottom panel, complemented strains with both native and constitutive RP27 promoters showed a loss of virulence on weeping lovegrass. Observations were consistent in 3 independent biological replicates (*n* = 3 plants). Scale bar represents 5 cm. **C)** Δpwl2 mutants display reduced pathogenicity on rice cultivar CO39. Conidial suspensions from Guy11, Δpwl2 (T6), and complemented Δpwl2(T6) + *PWL2*p:*PWL2* were used to inoculate 21-d-old seedlings of the blast-susceptible cultivar CO39, and disease symptoms were recorded after 5 dpi. The boxplots show the mean lesion density recorded per 5 cm **D)** and lesion area in mm^2^  **E)**. Data points of different colors represent different biological replicates. **F)** The Δpwl2 mutant exhibits enhanced pathogenicity on barley cultivar Baronesse (+*Mla3*). Conidial suspensions from Guy11 and Δpwl2 T6 were used to inoculate 10-d-old seedlings of barley lines Golden Promise (+*Mla8*), Siri (+*Mla8*), Nigrate (−*Mla3*), and Baronesse (+*Mla3*), and disease symptoms were recorded after 5 dpi. Conidial suspensions at 1 × 10^5^ mL^−1^ spores/mL were used for infection assays. **G)** The boxplot shows lesion size of barley seedlings infected with Guy11 and *Δpwl2* (T6) (individual points represent lesion area in mm^2^). The lower border and upper border of the box show the lower quartile and upper quartile, respectively. The line in the box shows the median. Significance between groups of samples in **D**, **E**, and **F)** were performed using unpaired Student's *t*-test with Welch correction. ****P < 0.0001, ***P < 0.001, **P < 0.01, *P < 0.05; NS, no significant difference. Error bars represent SEM in 3 independent biological replicates. Scale bar represents 5 mm.

We next set out to determine the function of *PWL2* in blast disease through gene functional analysis. Given that *PWL2* occurs in multiple copies, we used CRISPR/Cas9 genome editing (Foster et al. 2018) to simultaneously delete all 3 copies of *PWL2* found in Guy11. We designed a sgRNA to target *PWL2* and introduced a hygromycin phosphotransferase (*HPH*) encoding gene cassette (Fig. 4A). Transformants with ectopic integrations, or where some *PWL2* had not been deleted, gave a predicted PCR amplicon of 645 bp in size. By contrast, *Δpwl2* mutants generated a larger amplicon of 1.5 kb (Fig. 4A). Four putative transformants were selected for whole-genome sequencing. Raw reads were aligned to the *M. oryzae* reference genome using samtools v.15, before converting into BAM files and visualizing using IGV viewer. No reads mapping to the *PWL2* locus were identified in mutants T6 and T12, whereas fewer reads mapped to the same locus from sequenced T4 and T5 (Supplementary Fig. S5A). This confirmed that T6 and T12 are *Δpwl2* deletion mutants. Transformants T4, T5, and T6 displayed vegetative growth that was identical to Guy11 with normal dark concentric rings and light growing edges, while T12 showed reduced melanization and conidiation (Supplementary Fig. S5B). Given that *PWL2* is a host range determinant and avirulence gene, we reasoned that *M. oryzae Δpwl2* deletion mutants would gain virulence on weeping lovegrass (Kang et al. 1995; Sweigard et al. 1995) and barley cv. Baronesse expressing *Mla3* (Brabham et al. 2024). The *Δpwl2* deletion transformants T5, T6, and T12 and Guy11 were therefore used to infect weeping lovegrass seedlings and barley cv. Baronesse (+*Mla3*) using spray inoculation. The *Δpwl2* mutant T6 produced disease symptoms identical to those produced by *Eragrostis*-infecting isolate G17 and *Oryza*-infecting isolate Ina168 which lacks *PWL2,* while Guy11 and the 2 complemented isogenic strains (T6 + *PWL2*p:*PWL2*) and (T6 + *RP27*p:*PWL2*) were unable to cause disease (Fig. 4B). Similarly, T5 and T12 also produced disease symptoms on weeping lovegrass seedlings and cv. Baronesse (+*Mla3*) (Supplementary Fig. S5C). We conclude that T5, T6, and T12 are loss of recognition mutants of *PWL2* (Fig. 4B; Supplementary S5C), and we can be confident that T6 and T12 have complete deletion of all 3 copies of the gene.

We next tested whether Pwl2 has a virulence function during a compatible interaction between the blast fungus and its host. Having observed that T12 had slight differences in vegetative growth, we decided to select T6 for virulence assays and as a genetic complementation background for *PWL2*. T6 was used to inoculate susceptible rice cultivar CO39 by spray infection and on susceptible barley lines cv. Nigrate (-*Mla3*), cv. Siri (+*Mla8*), cv. Golden Promise (+*Mla8*), and resistant cv. Baronesse (+*Mla3*) using leaf drop inoculation. In 4 independent replicates, the *Δpwl2* mutant T6 showed a reproducible reduction in virulence on CO39 compared with wild-type Guy11 or a complemented isogenic strain (T6 + *PWL2*p:*PWL2*), based on lesion density and lesion size (Fig. 4, C to E). As expected, T6 was able to cause blast disease on cv. Baronesse (+*Mla3*) unlike Guy11 and produced statistically smaller lesion sizes on barley lines Siri (+*Mla8*) and Golden Promise (+*Mla8*), compared with Guy11. Both strains were able to infect Nigrate (−*Mla3*) without significant difference (Fig. 4, F and G). We conclude that *PWL2* contributes to the ability of *M. oryzae* to cause blast disease.

### Pwl2 suppresses host immunity

**Figure 5.**
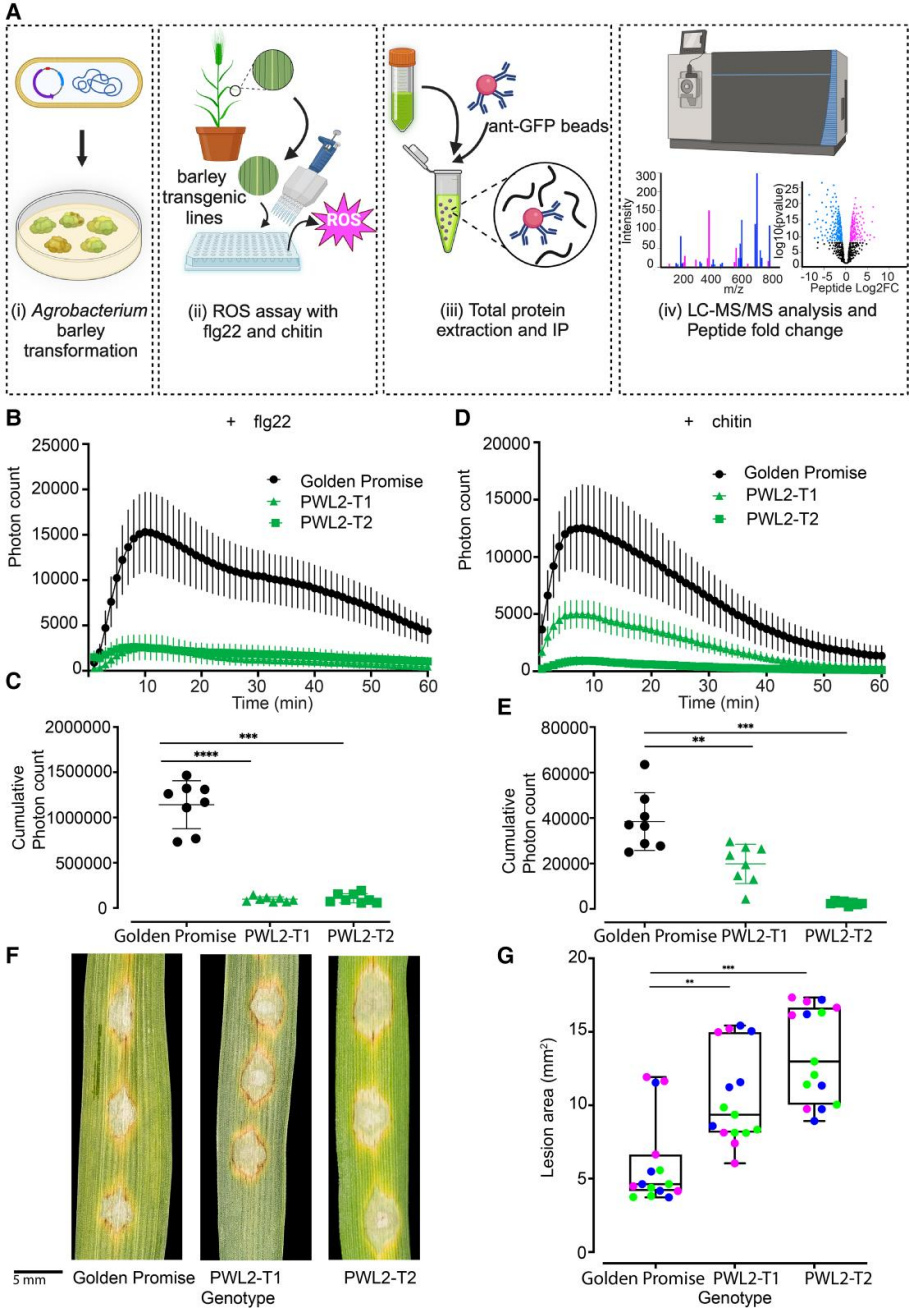
Pwl2 suppresses PAMP-induced ROS in transgenic barley lines. **A)** Schematic illustration to describe the workflow used to generate transgenic plants, test for PTI response, and identify putative Pwl2 interactors using discovery proteomics. ROS production in leaf disks collected from 4-wk-old 2 independent stable transgenic lines expressing Pwl2-YFP compared with cv. Golden Promise induced by 1 *μ*m flg22 **B** and **C)** or 1 mg/mL chitin **D** and **E)** (*n* > 8). For ROS assay, line graph **B** and **D)** points represent mean per time point and error bars represent SEM. Dot plots **C** and **E)** show integrated ROS production over 60 min; error bars represent mean ± Sd. The lower horizontal line shows the minimum value, the upper horizontal line shows the maximum value, and the middle line shows the mean value. **F)** Leaf drop infection on 2 independent barley transgenic lines expressing Pwl2-YFP compared with infection on wild-type cv. Golden Promise 3 to 4 dpi. Scale bar represents 5 mm. **G)** The boxplot represents the disease lesion area in mm^2^ on cv. Golden Promise compared with 2 independent Pwl2-YFP transgenic lines (data points of different colors represent different biological replicates). The lower border and upper border of the box show lower quartile and upper quartile range of the data, respectively. The line in the box shows the median. Unpaired Student's *t*-test with Welch correction was performed to determine significant differences: ****P < 0.0001, ***P < 0.001, **P < 0.01. Error bars represent SEM. These experiments were repeated 3 times to obtain consistent result.

Having determined that the Pwl2 effector contributes to virulence, we decided to study its biological function during host cell colonization. To do this, we first generated stable transgenic barley lines (in cv. Golden Promise) expressing *PWL2-YFP* (without its signal peptide) under control of the CaMV35S promoter (Fig. 5A). We tested 2 independent *PWL2-YFP* transgenic plants (Supplementary Fig. S6) for their response to 2 elicitors of pathogen-associated molecular patterns (PAMP)-triggered immunity (PTI), flg22 and chitin, compared with wild-type cv. Golden Promise (Fig. 5A). Barley perceives flg22 through the pattern recognition receptor FLS2 and chitin through HvCEBiP and HvCERK1, leading to immune responses such as generation of reactive oxygen species (ROS) (Yu et al. 2023 ). We observed that Pwl2 expression abolished flg22 (Fig. 5, B and C) and chitin-induced ROS generation (Fig. 5, D and E). Pwl2 therefore contributes to virulence by suppressing PTI during compatible interactions. To investigate the effect of elevated Pwl2 effector expression on blast infection, we infected 2 independent transgenic barley plants expressing *PWL2* with *M. oryzae* Guy11. *PWL2*-expressing barley lines were more susceptible and developed blast disease symptoms earlier (2 to 3 dpi) compared with infection of isogenic cv. Golden Promise (Fig. 5, F and G). We conclude that Pwl2 acts as a modulator of PTI that helps facilitate fungal infection.

### Pwl2 interacts with the heavy metal–binding isoprenylated protein HIPP43

**Figure 6.**
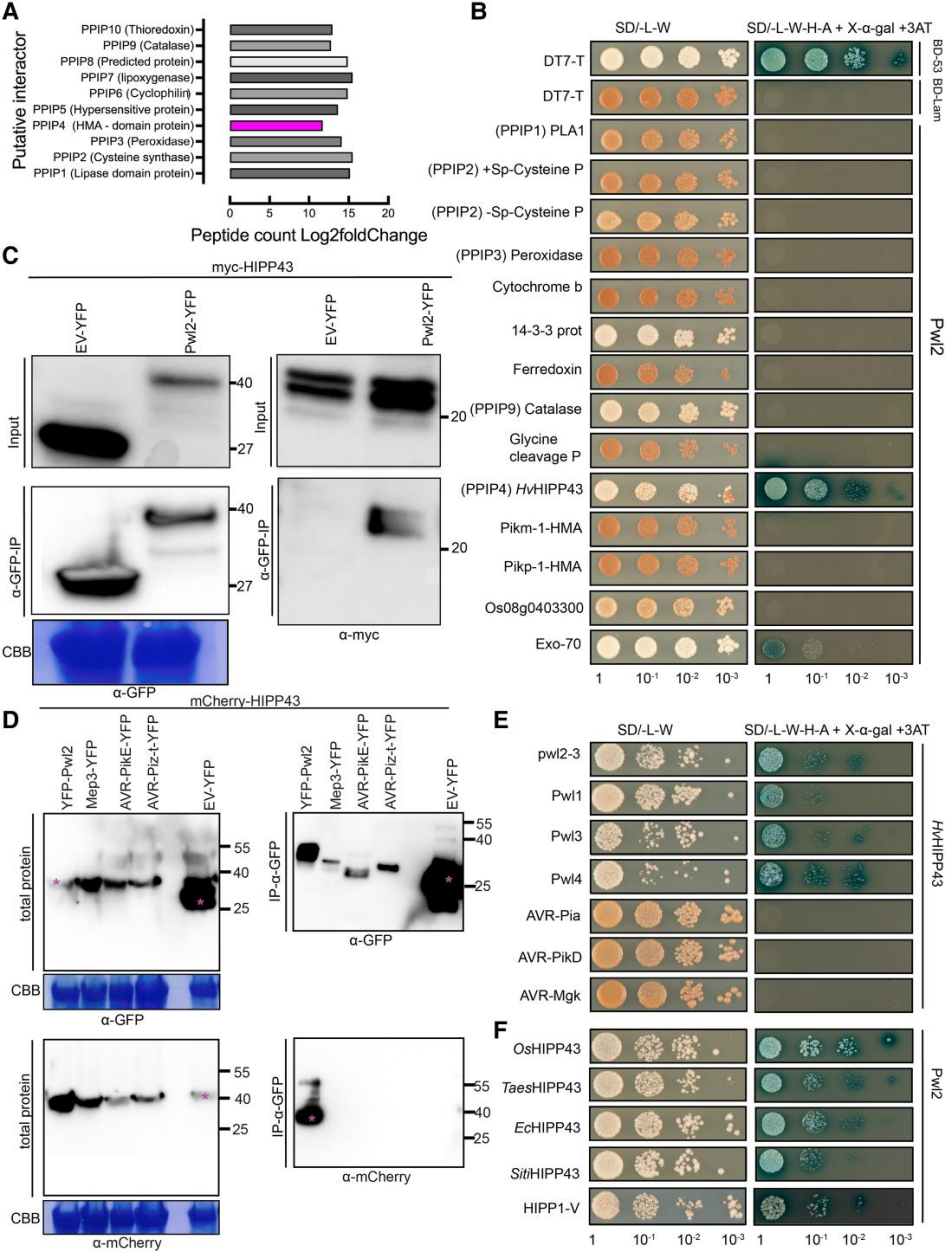
Pwl2 interacts with HIPP43 and its orthologs from other grass species. **A)** Putative Pwl2 interacting peptides were immunoprecipitated from protein extracts of 3-wk-old stable cv. Golden Promise transgenic lines expressing Pwl2-YFP or free cytoplasmic YFP using anti-GFP antibodies, and LC-MS/MS was performed to identify unique putatively interacting peptides. The scale 0 to 20 represents the Log2fold change in peptides when compared with the control. **B)** One-to-one yeast 2-hybrid between Pwl2 and selected top candidates from IP-MS analysis, PPIPs. HMA integrated in rice NLR Pikm-1 and Pikp-1 were used as specificity controls. Simultaneous cotransformation of pGADT7-Pwl2 (prey vector) together with pGBK-PPIPs (bait vector) or PGBKT7-53 and pGADT7-T (positive control) pGADTT7-T and pGBKT7-Lam (NC) into Y2H gold strain was carried out. Positive interaction resulted in the activation of 4 reporter genes and growth on high-stringency medium (−Ade, −Leu, –Trp, –His + X-α-gal and 3AT). Cotransformation also activates the expression of *MEL1*, which results in the secretion of α-galactosidase and the hydrolysis of X-α-gal in the medium, turning the yeast colonies blue. HIPP43 exclusively interacts with Pwl2 in SD/-L-W-H-A X-α-gal medium with 3AT added. These experiments were repeated several times over 3 yrs obtaining consistent result. **C)** Co-IP of Pwl2-YFP and Myc-HIPP43 or **D)** mCherry-HIPP43 in *N. benthamiana* leaves. C- or N-terminal GFP-tagged Pwl2 and C-terminal Myc-HIPP43 was cloned into the vector pGW514 and transformed into *Agrobacterium* strain *GV3101* and coinfiltrated into *N. benthamiana* leaves and left to incubate for 48 h. Immunoprecipitates were obtained with anti-GFP affinity matrix beads and probed with anti-GFP-peroxidase, anti-mCherry-peroxidase, and anti-Myc-peroxidase, Horseradish Peroxidase (HRP)-conjugated antibodies. Total protein extracts were also probed with appropriate (HRP-conjugated) antibodies. Magenta asterisks indicate expected band sizes. **E)** Y2H analysis shows in upper panel, *Hv*HIPP43 interacts with *PWL* gene family products Pwl1, Pwl3, Pwl4, and variant *pwl2-3*. **F)** Pwl2 interacts with HIPP43 orthologs from rice (*Os*HIPP43), wheat (*Taes*HIPP43), weeping love grass (*Ec*HIPP43), foxtail millet (*SitiHIPP43*), and wild wheat (HIPP1-V). These experiments were repeated several times over 3 yrs obtaining consistent result.

To determine the likely target of Pwl2, transgenic barley lines expressing Pwl2-YFP were used in co-immunoprecipitation (co-IP) coupled to MS (IP-MS) analysis. This was followed by spectral search in the *H. vulgare* (barley, cv. Morex version 3) proteome database. We identified a total of 282 frequently occurring proteins in the 8 biological replicates containing Pwl2, which were not identified in the 6 free-YFP control samples. To avoid focusing on false positives in the form of sticky and abundant proteins, fold changes were calculated by first determining the average number of peptides per protein candidate, before estimating log_2_ fold change compared with the control experiment. We further filtered for proteins that produced at least 2 peptide hits in more than half of replicates (≥4/8 biological replicates) (Supplementary Data Set 3). A total of 52 proteins met this criterion and were considered as enriched in samples compared with controls and were therefore selected for further analysis. Furthermore, because Pwl2 attenuates the ROS burst in barley transgenic lines, we focused initially on protein candidates previously reported to have potential roles in immunity or ROS generation (Fig. 6A). These were selected for one-to-one interactions using a yeast-2-hybrid (Y2H) assay by cotransforming constructs expressing Pwl2 and putative Pwl2 interacting proteins (PPIPs) identified by IP-MS (Fig. 6A). Pwl2 showed a strong interaction with PPIP4, a HMA domain protein (Fig. 6B). We named this protein *Hv*HIPP43, for *Hordeum vulgare* heavy metal domain containing isoprenylated plant protein 43 based on homology to *Os*HIPP43 (Zdrzałek et al. 2024). We also tested to ensure that neither Pwl2 nor HIPP43 demonstrated auto-activity in Y2H drop out media (Supplementary Fig. S7A). To independently verify the interaction between Pwl2 and HIPP43, we carried out co-IP analysis using protein extracts from *Nicotiana benthamiana* and confirmed that *Hv*HIPP43 interacts with Pwl2 (Fig. 6, C and D). Conversely, 3 other MAX-fold effectors, MEP3, AVR-PikE, AVR-Piz-t, or free-YFP did not interact with HIPP43 (Fig. 6D). We next tested whether *PWL* gene family–encoded proteins Pwl1, Pwl3, Pwl4, and the pwl2-3 variant could also interact with *Hv*HIPP43 and showed that they can all interact based on Y2H assays, suggesting that Pwl2 belongs to an effector family that interacts with a host sHMA protein (Fig. 6E). By contrast, 3 MAX-fold effectors AVR-Pia, AVR-PikD, and AVR-Mgk did not interact with HIPP43 (Fig. 6E). Barley and wheat have 3 copies of HIPP43 per haploid genome, but based on our IP-MS results (Supplementary Data Set 4), we identified peptides that mapped to 2 copies of HIPP43, which both strongly interacted with Pwl2 in Y2H assays HIPP43 (Supplementary Fig. S7, B and C).

### Pwl2 can interact with HIPP43 orthologs of diverse grass species

HIPPs are expanded in plant genomes and to test whether HIPP43 has orthologs in other grass species, we generated a maximum likelihood phylogenetic tree of HMA domains from diverse grasses with high-quality genomes and annotations (Supplementary Fig. S7D). The HIPP43 family formed a distinct clade which includes orthologs from rice (*O. sativa)*, wheat (*T. aestivum*), wild wheat (*Haynaldia villosa*), foxtail millet (*Setaria italica*), weeping lovegrass (*E. curvula*), Sorghum (*Sorghum bicolor*), *Oropetium thomaeum*, *Zea mays*, and *Brachypodium distachyon* (Supplementary Fig. S7D). Furthermore, we could identify copy number variation ranging from one to 5 copies in grasses, with barley and wheat (i.e. *Triticeae* lineage) having 3 or more paralogs of HIPP43, one per haploid genome (Supplementary Fig. S8A). We found that Pwl2 interacts with *Hv*HIPP43 orthologs from rice (*Os*HIPP43), *Triticum* (*Taes*HIPP43), *Setaria* (*SitiHIPP43*), *Eragrostis* (*Ec*HIPP43), and *Haynaldia* (HIPP1-V) in Y2H assays (Fig. 6F). The interaction of Pwl2 with rice *Os*HIPP43 was verified by in vitro biochemistry and a structure of the complex was obtained by X-ray crystallography (Zdrzałek et al. 2024). We also analyzed the transcriptional profile of *Os*HIPP43 during infection of Guy11 in a susceptible rice line CO39 (Yan et al. 2023) and found that *Os*HIPP43 expression is upregulated during plant infection, consistent with a role in host defense (Supplementary Fig. S8B).

### Overexpression of HIPP43 suppresses PTI and increases blast susceptibility

**Figure 7.**
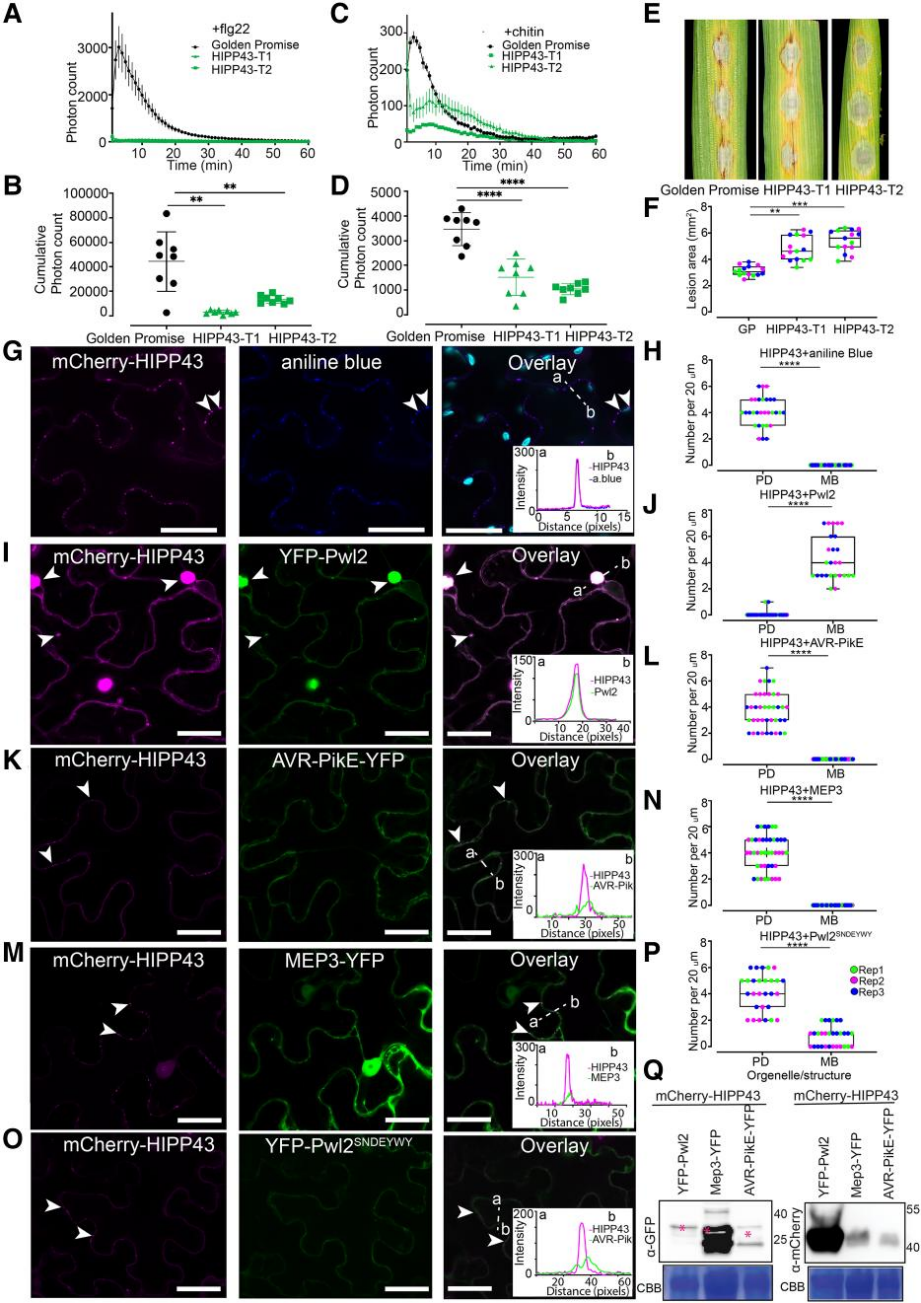
*Hv*HIPP43 suppresses PAMP-induced ROS in transgenic barley and is stabilized by Pwl2. **A** to **D)** ROS production measured from leaf disks collected from 4-wk-old stable transgenic line expressing YFP-HIPP43 and cv. Golden promise (control) in the absence and presence of 1 *μ*m flg22 or 1 mg/mL Chitin (*n* > 8). Line graph **A** and **C)** points represent mean per time point, and error bars represent SEM. Dot plots **B** and **D)** show integrated ROS production over 60 min, and error bars represent mean ± Sd. The lower horizontal line shows the minimum value, the upper horizontal line shows the maximum value, and the middle line shows the mean value. **E)** Leaf drop infection on barley transgenic lines expressing YFP-HIPP43 compared with wild-type Golden Promise. Conidial suspensions at 1 × 10^5^ mL^−1^ spores/mL from Guy11 were used for inoculation. Disease symptoms were recorded after 4 dpi. Scale bar represents 5 mm. **F)** Box plots show lesion area in mm^2^. All experiments were repeated 3 times giving consistent results. **G** and **H)** Micrographs and boxplot showing mCherry-HIPP43 localizing as small puncta on the plasma membrane when expressed in *N. benthamiana.* White arrowheads indicate regions of HIPP43 localization at the PD. Staining of callose using aniline blue overlaps with mCherry-HIPP43, confirming mCherry-HIPP43 localizes exclusively at the PD localization and cytoplasmic mobile bodies (MBs) localization is absent. **I** and **J)** Micrographs and boxplot showing the presence of YFP-Pwl2, alters mCherry-HIPP43 localization, which was observed to translocate to the cytoplasm, as mobile cytoplasmic bodies. White arrowheads indicate cytoplasmic puncta of HIPP43 and Pwl2 colocalization. **K** and **L)** Micrographs and boxplot showing mCherry-HIPP43 remains in the PD in the presence of cytoplasmically localized AVR-PikE. **M** and **N)** Micrographs and boxplot showing mCherry-HIPP43 remains in the PD in the presence of cytoplasmically and PD localized MEP3. **O** and **P)** Micrographs and boxplot showing mCherry-HIPP43 remains in the PD in the presence of Pwl2^SNDEYWY^, a mutant that does not interact with HIPP43. For **K**, **M**, and **O)**, white arrowheads indicate regions of HIPP43 localization at the PD. Dotted lines in the overlay panels correspond to distance and direction of line intensity plot. Scale bars represent 20 *µ*m. To determine colocalization, 50 or more transformed cells were counted per replicate. For box plots, the lower horizontal line shows the minimum value, the upper horizontal line shows the maximum value, and the middle line shows the median value. Data points of different colors represent different biological replicates. Unpaired Student's *t*-test was performed to determine significant differences ****P < 0.0001, ***P < 0.001, **P < 0.01. Error bars represent SEM. Microscope imaging experiments were repeated several times over 2 yr with consistent results. **Q)** Western blot to show that Pwl2 does not degrade HIPP43. Total protein extracts from *N. benthamiana* leaves coinfiltrated with YFP-Pwl2, MEP3 or AVR-PikE, and mCherry-HIPP43 were immunoblotted and probed with anti-GFP-peroxidase (left) or anti-mCherry-peroxidase (right) (HRP-conjugated) antibodies. Magenta asterisks indicate expected band sizes.

To investigate the role of the Pwl2–HIPP43 interaction, we generated barley transgenic lines overexpressing *Hv*HIPP43. Two independent transgenic lines showing strong expression (Supplementary Fig. S8C) were challenged with 2 PTI elicitors, flg22 (Fig. 7, A and B) and chitin (Fig. 7, C and D). Strikingly, we observed that both the chitin and flg22-induced ROS burst was abolished or reduced in plants expressing *Hv*HIPP43, compared with wild type (Fig. 7, A to D). Similarly, when we challenged transgenic barley plants expressing *Hv*HIPP43 with *M. oryzae*, we observed increased susceptibility compared with the wild-type cv. Golden Promise, and disease lesions appeared earlier than in wild-type plants (Fig. 7, E and F). These findings are consistent with overexpression of both *PWL2* and HIPP43 leading to enhanced blast disease susceptibility.

### Pwl2 prevents plasmodesmatal localization of HIPP43

To investigate the function of *Hv*HIPP43, we performed live-cell imaging following transient expression in *N. benthamiana* to determine its subcellular localization. When tagged at the N-terminus (mCherry-HIPP43), *Hv*HIPP43 localized predominantly to puncta at the plasma membrane that colocalize with callose staining (aniline blue), consistent with sites of PD (Fig. 7, G and H). Like all HIPPs, *Hv*HIPP43 has an isoprenylation motif at its C-terminus (Supplementary Fig. S9, A and B), implicated in membrane anchoring (Hála and Žárský 2019), so we investigated whether PD localization is affected by its deletion. We observed that PD localization was indeed abolished in the absence of the isoprenylation motif and -Iso-mCherry-HIPP43 (*Hv*HIPP43 without isoprenylation motif) instead accumulated in the nucleoplasm and at the plasma membrane (Supplementary Fig. S9C). Moreover, coexpression with a plasma membrane marker LTi6b-GFP and aniline blue staining confirmed its localization at the plasma membrane and loss of PD localization (Supplementary Fig. S9C) compared with the wild type (Supplementary Fig. S9D).

Given the strong interaction between Pwl2 and HIPP43 and the localization of HIPP43 to PD, we were keen to see the effect of coexpressing the effector and putative host target in the same cells. When we coexpressed YFP-Pwl2 with mCherry-HIPP43, they colocalized in the cytosol as mobile puncta approximately 2 *μ*m in diameter, away from PD (Fig. 7I). In some cases, we observed larger mobile structures ranging from 2 to 4 *μ*m in diameter (Fig. 7I). We also tested whether Pwl1, Pwl3, and Pwl4 (Supplementary Fig. S10A) could also colocalize with HIPP43 when coexpressed in *N. benthamiana.* In the presence of Pwl1, HIPP43 PD localization was abolished, and the 2 proteins colocalize in the cytoplasm and partially in cytoplasmic mobile structures (Supplementary Fig. S10B) Conversely, Pwl3 did not affect mCherry-HIPP43 localization to PD (Supplementary Fig. S10C), while Pwl4 (Supplementary Fig. S10D) only showed cytoplasmic colocalization without larger mobile bodies forming. In addition, because we had observed that *Hv*HIPP43 orthologs from wheat (*Taes*HIPP43), foxtail millet (*Siti*HIPP43), and weeping lovegrass (*Ec*HIPP43) interact with Pwl2 in Y2H assays, we also tested whether *Taes*HIPP43, *Siti*HIPP43, or *Ec*HIPP43 localize as PD puncta in the same way as *Hv*HIPP43. Interestingly, *Taes*HIPP43 (wheat) mostly localized to the nucleus, cytoplasm and PD, even though PD localization was reduced (Supplementary Fig. S10E), whereas *Ec*HIPP43 (weeping lovegrass) (Supplementary Fig. S10F) and *Siti*HIPP43 (foxtail millet) (Supplementary Fig. S10G) localized to small puncta equivalent to PD, like *Hv*-HIPP43. Because Pwl2 is able to alter subcellular localization of *Hv*HIPP43, we tested whether this also occurred when *Siti*HIPP43 and Pwl2 were coexpressed. We observed Siti-HIPP43/Pwl2 colocalization as cytoplasmic mobile bodies away from PD, mirroring the Pwl2/HvHIPP43 interaction (Supplementary Fig. S10, H to J). When considered together, we conclude that Pwl2 and its family members can interact with *Hv*HIPP43 and that Pwl2 can also interact with HIPP43 orthologs from other cereals, thereby altering their deployment to PD.

### Pwl2 consistently alters the plasmodesmatal localization of HIPP43

To understand the effect of Pwl2 expression on the PD localization of *Hv*HIPP43, we carried out a detailed quantitative analysis. In control experiments we expressed *Hv*HIPP43 with free cytoplasmic YFP, and 2 MAX effectors AVR-PikE and MEP3 (Yan et al. 2023). In 6 independent coexpression experiments, we observed colocalization of Pwl2 and *Hv*HIPP43 as mobile cytoplasmic puncta or large cytoplasmic mobile structures, while PD localization was significantly reduced (Fig. 7, I and J). By contrast, colocalization of *Hv*HIPP43 with AVR-PikE or MEP3 led to mCherry-*Hv*HIPP43 fluorescence remaining at PD (Fig. 7, K to N). Moreover, mCherry-HIPP43 fluorescence showed greater intensity when coexpressed with Pwl2 compared with when coexpressed with AVR-PikE or MEP3 (Fig. 7, K to N). It is therefore possible that Pwl2 is able to stabilize *Hv*HIPP43 or increases its accumulation, because the mCherry-*Hv*HIPP43 signal is barely detectable in the absence of Pwl2 (Fig. 7, K and M). Our previous structural analysis demonstrated that Pwl2 and *Os*HIPP43 produce a robust binding interface that requires up to 7 mutations in Pwl2 (*Pwl2*^SNDEYWY^) to abolish (Zdrzałek et al. 2024). To test whether binding of Pwl2 to HIPP43 is necessary for the alteration in its cellular localization, we coinfiltrated mCherry-HIPP43 and YFP-Pwl2^SNDEYWY^ in *N. benthamiana* and carried out live-cell imaging. We were able to observe YFP-Pwl2^SNDEYWY^ expression, but this did not alter mCherry-*Hv*HIPP43 localization at PD (Fig. 7, O to P). Furthermore, the fluorescence intensity of mCherry-*Hv*HIPP43 did not increase as it did upon coexpression with YFP-Pwl2 (Fig. 7, I and O). To rule out the possibility that Pwl2 degrades *Hv*HIPP43 upon interaction in plant cells, we carried out immunoblot analysis using protein extracts from *N. benthamiana* following coinfiltration of mCherry-HIPP43 with Pwl2, MEP3, or AVR-PikE. Strikingly, the presence of Pwl2 led to pronounced mCherry-*Hv*HIPP43 accumulation compared with when coinfiltrated with MEP3 or AVR-PikE (Fig. 7Q). We were also concerned that Pwl2/HIPP43 colocalization as puncta could be mis-interpreted as also occurring at PD. To rule out such a possibility, we stained PD with aniline blue after coinfiltrating *Hv*HIPP43 with free-YFP, YFP-Pwl2, AVR-PikE-YFP, and MEP3-YFP. In a control experiment, we expressed free-YFP, YFP-Pwl2, AVR-PikE-YFP, and MEP3-YFP in the absence of *Hv*HIPP43. We found that free-YFP, Pwl2, AVR-PikE-YFP, and MEP3 show localization to the cytosol (Supplementary Fig. S11, A to D), but MEP3-YFP also showed some additional PD localization (Supplementary Fig. S11D). By contrast, YFP-Pwl2 was always observed as cytoplasmic mobile puncta (Supplementary Fig. S11B). When cells expressing Pwl2 were stained with aniline blue there was no colocalization of Pwl2 and the aniline blue signal (Supplementary Fig. S11, E to G). In the presence of mCherry-HIPP43 and YFP-Pwl2, colocalization at cytoplasmic puncta/mobile bodies was observed and did not colocalize with aniline blue staining (Supplementary Fig. S11H). In limited cases where colocalization was observed, this was transient because the Pwl2/HIPP43 puncta are mobile. By contrast, when expressed with 2 MAX-fold effectors AVR-PikE and MEP3, or free-YFP, mCherry-HIPP43 colocalized with aniline blue to PD (Supplementary Fig. S11, I to K). In addition, when YFP-Pwl2 was coexpressed with a nonfluorescently tagged HIPP43 (myc-HIPP43), we could still observe formation of small and large cytoplasmic mobile bodies, ruling out any interference by the fluorescent tag (Supplementary Fig. S12). To investigate the motility of HIPP43-Pwl2 puncta, we carried out time-lapse imaging using confocal laser microscopy of aniline blue–stained *N. benthamiana* cells following coinfiltration of mCherry-HIPP43 with YFP-Pwl2, MEP3-YFP, AVR-PikE-YFP, or YFP-Pwl2^SNDEYWY^. Blue-stained puncta equivalent to PD remained immobile, while mCherry-HIPP43/YFP-Pwl2 fluorescence was observed at mobile cytoplasmic structures (see Supplementary Video S1 and individual frames shown in Supplementary Fig. S13A). By contrast, colocalization of mCherry-HIPP43 at aniline blue-stained PD was clearly visible and not altered by the presence of 2 MAX-fold effectors MEP3-YFP and AVR-PikE-YFP, YFP-Pwl2^SNDEYWY^, or of free-YFP (see Supplementary Videos S2 to S5 and frames in Supplementary Fig. S13, B to E). When considered together, these results provide evidence that Pwl2 interacts with HIPP43 altering its cellular location away from PD.

### The ability of Pwl2 to interact with HIPP43 is necessary for its avirulence and virulence functions

**Figure 8.**
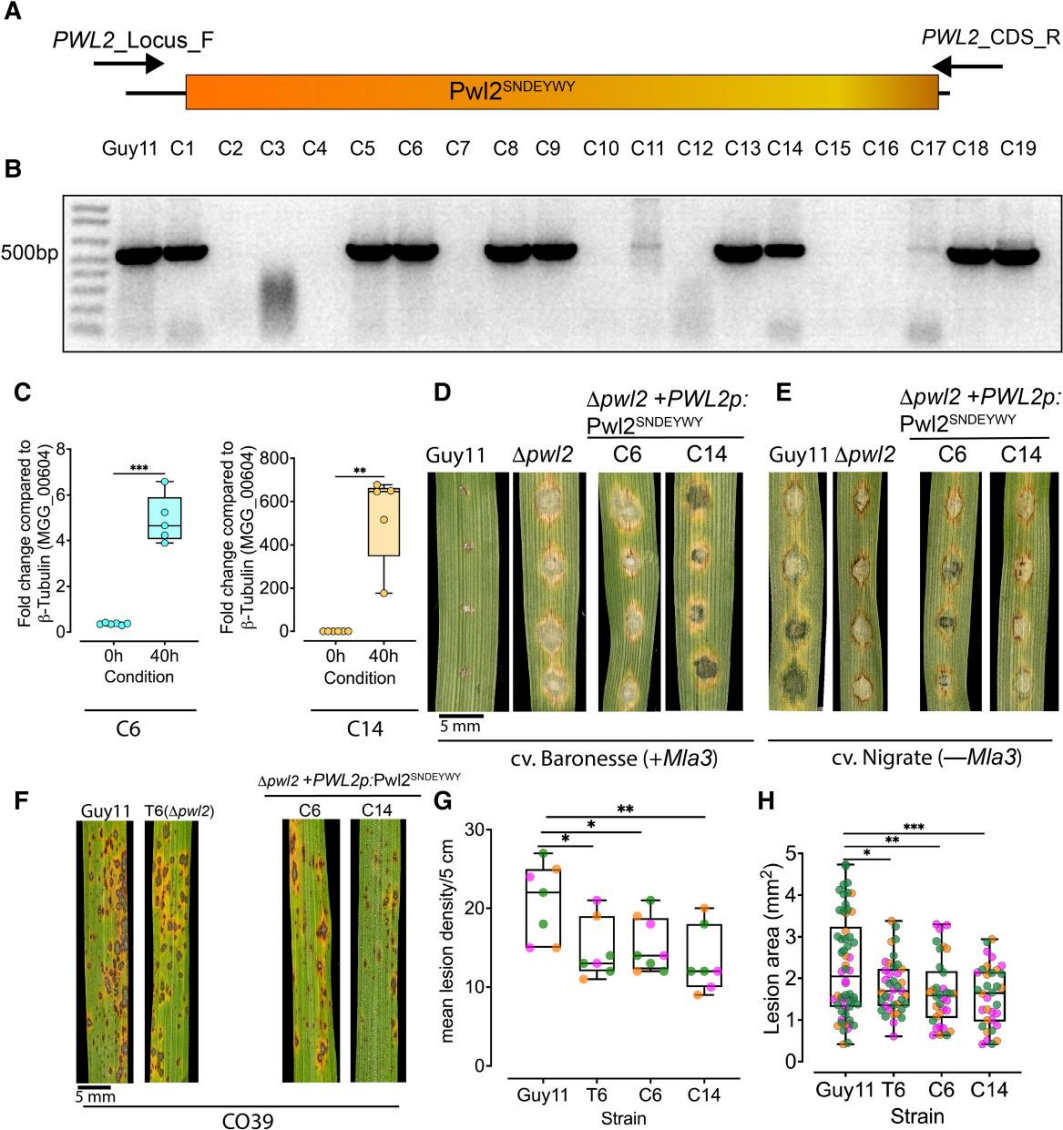
Pwl2^SNDEYWY^ does not complement Mla3 recognition and virulence on a blast-susceptible rice cultivar CO39. **A** and **B)** Schematic representation to show how complemented Δpwl2 + *PWL2*p:*Pwl2^SNDEYWY^*-positive transformants were screened using PCR to amplify *PWL2* coding sequence. **C)** Boxplots showing relative expression as log2 fold change of *Pwl2^SNDEYWY^* in 2 selected transformants, C6 (left panel) and C14 (right panel) using qRT-PCR. The lower border and upper border of the box show lower quartile and upper quartile range of the data, respectively. The line in the box shows the median. Unpaired Student's *t*-test with Welch correction was performed to determine significant differences. Detached leaves of 10-d-old seedlings of barley were inoculated with Δpwl2 + *PWL2*p:*Pwl2^SNDEYWY^*, and infected tissue was collected 40 hpi and used for RNA isolation (3 biological replicates); cDNA was synthesized and samples were used for qRT-PCR. **D** and **E)** Δpwl2 + *PWL2*p:*Pwl2^SNDEYWY^*-complemented strains C6 and C14 produced compatible disease lesions on barley cultivar Baronesse (+*Mla3*) **D)** and Nigrate (−*Mla3*) **E)**. Conidial suspensions at 1 × 10^5^ mL^−1^ spores/mL from Guy11, Δpwl2, and complemented Δpwl2 + *PWL2*p:*Pwl2^SNDEYWY^* were used to inoculate 10-d-old seedlings of barley, and disease symptoms were recorded after 5 dpi. **F** to **H)** Complemented Δpwl2 + *PWL2*p:*Pwl2^SNDEYWY^* display reduced pathogenicity on rice cultivar CO39. Mean lesion density recorded per 5 cm **G)** and lesion area in mm^2^  **H)** of Guy11 compared with Δpwl2 and complemented Δpwl2 + *PWL2*p:*Pwl2^SNDEYWY^* following leaf spray infection. Box and whisker plots with individual data points from leaves collected in 3 independent replicates. Error bars represent SEM. Data points of different colors represent different biological replicates. Conidial suspensions at 1 × 10^5^ mL^−1^ spores/mL from Guy11, Δpwl2 T6, and complemented Δpwl2 + *PWL2*p:*Pwl2^SNDEYWY^* were used to inoculate 21-d-old seedlings of the blast-susceptible cultivar CO39, and disease symptoms were recorded after 5 dpi. The lower border and upper border of the box show lower quartile and upper quartile range of the data, respectively. The line in the box shows the median. Unpaired Student's *t*-test with Welch correction was performed to determine significant differences. **P < 0.01, *P < 0.05; NS, no significant difference. Scale bars represent 5 mm.

As the interaction between Pwl2 and HIPP43 appears to be critical for altering its subcellular localization to PD, we decided to test if this was also necessary for the role of Pwl2 in blast disease. We therefore set out to see whether the *pwl2*^SNDEYWY^ allele could complement the mutant phenotypes of a *M. oryzae Δpwl2* mutant. To do this, we transformed *Δpwl2* mutant with a construct expressing Pwl2^SNDEYWY^ under its native promoter (*PWL2p:pwl2^SNDEYWY^*) and successful transformants were selected (Fig. 8, A and B). We then quantified *Pwl2^SNDEYWY^* gene expression in selected transformants using qRT-PCR (Fig. 8C). We reasoned that if recognition of Pwl2 by Mla3 requires HIPP43 recognition (Gómez De La Cruz et al. 2024), then Pwl2^SNDEYWY^ should not complement the observed gain of virulence of *Δpwl2* mutants on barley cv. Baronesse (+*Mla3*). Moreover, if the Pwl2/HIPP43 interaction is required for the virulence function of Pwl2, then Pwl2^SNDEYWY^ should not restore virulence to *Δpwl2* mutants. In 3 independent experiments of 2 independent strains, C6 (*Δpwl2* + *PWL2p:pwl2^SNDEYWY^*) and C14 (*Δpwl2* + *PWL2p:pwl2^SNDEYWY^*) showed a compatible interaction on cv. Baronesse (+*Mla3*) (Fig. 8D) providing evidence that the Pwl2^SNDEYWY^ is not recognized by Mla3. The 2 strains C6 and C14 still retained the ability to infect a susceptible barley cv. Nigrate (−*Mla3*) (Fig. 8E). We also observed that *Δpwl2* + *PWL2p:Pwl2^SNDEYWY^* transformants C6 and C14 did not restore full virulence on susceptible rice cv. CO39 compared with the wild-type Guy11 (Fig. 8F), based on lesion density (Fig. 8G) and lesion size (Fig. 8H). We conclude that the interaction between Pwl2 and HIPP43 is required for both its recognition as an avirulence factor and its function as a virulence determinant.

## Discussion

The Pwl2 effector was first identified as a host specificity determinant for infection of the forage grass species weeping lovegrass (Kang et al. 1995; Sweigard et al. 1995). Its initial identification provided evidence that host range in plant pathogenic fungi was conditioned in a similar way to cultivar specificity in a gene-for-gene manner, involving dominant pathogen genes recognized by the products of cognate disease resistance genes (Kang et al. 1995). Furthermore, Pwl2 was found to belong to an expanded gene family, suggesting an important function in pathogenicity and fitness (Kang et al. 1995). However, in the following 2 decades, the function of Pwl2 has remained elusive despite its extensive use as a marker in cell biological studies for investigating effector regulation, secretion, and delivery during plant infection (Kankanala et al. 2007; Giraldo et al. 2013; Oliveira-Garcia et al. 2023).

In this study, we set out to explore the function of Pwl2 and investigate why it is such a highly conserved effector in *M. oryzae*. We found that *PWL2* is highly conserved in *M. oryzae*, including each of its host-limited forms and even in sister *Magnaporthe* species infecting crabgrass and pearl millet. The observation that *PWL2* and the wider *PWL* family genes have been maintained in the global blast population with effector variants in certain host-adapted lineages, suggests that Pwl2 and members of this family serve an important function in pathogenesis. Furthermore, *PWL2* is present at a high copy number in many *M. oryzae* isolates having undergone extensive gene duplication and is specifically expressed during the initial stages of blast infection, particularly at the stage when *M. oryzae* moves from an initially colonized epidermal cell, following appressorium penetration, to adjacent host cells. This cell-to-cell movement by the fungus utilizes PD-containing pit fields and the fungus forms a transpressorium structure that undergoes severe hyphal constriction to traverse each pit field (Kankanala et al. 2007; Cruz-Mireles et al. 2021), a process that requires activity of the Pmk1 MAP kinase (Sakulkoo et al. 2018). We found that *PWL2* is expressed during this process in a Pmk1-dependent manner, forming part of a regulated set of effectors deployed by the fungus during cell-to-cell movement. The generation of a *Δpwl2* mutant, made possible by CRISPR/Cas9 gene editing to delete all 3 native copies of the gene, enabled us to confirm the role of Pwl2 in host specificity to weeping lovegrass (Sweigard et al. 1995), as an avirulence effector for barley Mla3 (Brabham et al. 2024), and also revealed its importance in blast disease. Pwl2 is therefore an important virulence determinant for blast disease, explaining its conservation and amplification.

To identify the likely target of Pwl2, we utilized discovery proteomics, which revealed its interaction with an isoprenylated small HMA protein, HIPP43. This is consistent with Pwl2 being a MAX effector—many of which have been shown to interact with sHMA protein domains—although previous studies have focused on incorporation of sHMA domains into paired NLR immune receptors leading to disease resistance (Ortiz et al. 2017; Bentham et al. 2021; Maidment et al. 2021; Mukhi et al. 2021). The crystal structure of the Pwl2/HIPP43 complex (Zdrzałek et al. 2024) demonstrates that Pwl2 uses an expansive interface to mediate binding to HIPP43, largely using elements of the MAX-fold to interact with the β-sheet of its host target. Indeed, when HIPP43 is incorporated into the Pik-1 NLR, in place of its naturally occurring integrated HMA domain, this leads to an immune response to Pwl2 (Zdrzałek et al. 2024). A recent study has also revealed that the barley resistance protein Mla3 acquired the ability to bind Pwl2 by mimicking the HMA fold of its host target, HIPP43 (Gómez De La Cruz et al. 2024). Interestingly, polymorphic residues found in Pwl2 variants, such as pwl2-2 and pwl2-3 are not located at the HIPP43 binding interface but are located away from the MAX-fold in the C-terminal helix of Pwl2. It is possible that these polymorphic residues are essential to stabilize the Pwl2, MAX-fold/HMA-like interface in Mla3. Consistent with this idea, introducing mutations that disrupt the Pwl2/HIPP43 interaction also results in loss of recognition by Mla3.

In spite of the importance of sHMA domains in plant immunity, little is known regarding their function or why they are targeted by fungal effectors. HMA domains are known to be involved in biotic/abiotic stress responses, transport of metals, and metal detoxification, and their expression can be organ-specific or tissue-specific within roots, leaves, or stems (Barth et al. 2009; Zschiesche et al. 2015; Zhang et al. 2020). Moreover, sHMAs can localize to the nucleus, plasma membrane, cytoplasm, or to PD (Barth et al. 2009; Cowan et al. 2018; Barr et al. 2023; Oikawa et al. 2024). Furthermore, sHMAs are expanded in plant genomes with more than 45, and more than 50, sHMA domain-encoding genes occurring in *Arabidopsis* and rice, respectively (de Abreu-Neto et al. 2013). Unique intragenic deletions in OsHIPP05 (*Pi21*), a proline-rich HMA domain protein-encoding gene in rice leads to rice blast resistance (Fukuoka et al. 2009) and gene silencing or deletion mutants of *TaHIPP1* or *AtHMAD1* in wheat and *Arabidopsis* provide enhanced resistance against *Puccinia striiformis* f. sp*. tritici* and *Pseudomonas syringae* DC3000, respectively (Imran et al. 2016; Wang et al. 2023). In addition, deletion mutants of *AtHIPP27* in *Arabidopsis* lead to increased resistance against cyst nematodes (Radakovic et al. 2018). However, it is not clear why deletion of sHMA protein-encoding genes impacts immunity. Therefore, the observation that Pwl2 interacts with HIPP43 is revealing, especially because overexpression of either Pwl2 or *Hv*HIPP43 suppresses PTI responses and enhances blast disease susceptibility. How this potential enhancement of HIPP43 activity is stimulated by Pwl2 is not completely clear, but transient coexpression of both Pwl2 and HIPP43 sequesters HIPP43 away from PD, and the 2 proteins instead colocalize in large mobile structures within the cytoplasm. The localization of HIPP43 to PD requires its isoprenylation motif and may be associated with an immune signaling role at this site. Given the Pmk1 MAP kinase–dependent expression of Pwl2 during PD traversal, it may be that sequestering HIPP43 away from these sites is critical for the fungus to invade plant tissue efficiently. This is consistent with the reduced virulence phenotype of *Δpwl2* mutants, which results in slower generation of disease symptoms and reduced lesion size. A recent study has highlighted how a *P. infestans* effector PiE354 can interfere with a host immune response by re-routing a plant Rab8a away from the plant/pathogen interface, a similar potential effector function that alters the cellular location of a target rather than impairing a particular function (Yuen et al. 2024).

Localization of 2 HIPP proteins, HIPP7 and HIPP26, to PD has been previously reported, which is also dependent on isoprenylation motifs like HIPP43 (Cowan et al. 2018; Guo et al. 2021). Although characterized HIPPs have been proposed to be metallochaperones, there are no studies that link metal detoxification to immunity. Some studies suggest that there may be a connection between concentration of metal ions, such as iron, copper, cadmium, and zinc, with plasmodesmatal permeability (O’Lexy et al. 2018), which might explain the function of a plasmodesmatal localized HIPP in regulating permeability, associated with their role in immunity. Both the HMA domain and isoprenylation regions of HIPPs have been shown to be important for plant immunity. For example, a mutation targeting the proline-rich region of *pi21* is sufficient to lead to gain of resistance against *M. oryzae* (Fukuoka et al. 2009), while interfering with the isoprenylation of HIPP1-V from wild wheat (*H. villosa*) leads to loss of resistance against *Blumeria graminis* f. sp. *tritici* accompanied by reduced HIPP1-V localization to plasma membrane. Interestingly, HIPP1-V interacts with the E3-ligase CMPG1-V at the plasma membrane leading to resistance to powdery mildew in an isoprenylation-dependent manner (Wang et al. 2023). This interaction has been reported to activate expression of genes involved in ROS generation and salicylic acid biosynthesis, suggesting that HIPP1-V is required for PTI regulation (Wang et al. 2023). Interestingly, we found that HIPP1-V is an ortholog of HvHIPP43 and can interact with Pwl2 in a Y2H assay. It is possible, therefore, that the Pwl2 interaction with HvHIPP43 attenuates PTI through a similar mechanism, which will require further investigation.

How the change in cellular localization of HIPP43 induced by the Pwl2 effector prevents its function in immunity or leads to a new role that enhances disease susceptibility remains unclear. A very recent study has provided evidence that *M. oryzae* AVR-Pik binding stabilizes the rice sHMA proteins OsHIPP19 and OsHIPP20 (Oikawa et al. 2024), suggesting that the function of Pwl2 may be mirrored by other blast effectors, targeting a wider pool of HIPPs. In this regard, future work will be necessary to determine whether HIPP43 directly regulates ROS generation, which might reduce permeability of PD (Cui and Lee 2016), or acts in a more indirect manner through interaction with other signaling components involved in PTI. Finally, Pwl2 is known to be a highly mobile effector and has been shown to move into neighboring rice cells ahead of *M. oryzae* hyphal growth (Giraldo et al. 2013). This has been suggested to be a step to prepare un-invaded cells for fungal colonization, consistent with its Pmk1-dependent regulation. Pwl2 may therefore alter the subcellular localization and concentration of HIPP43, sequestering it away from PD that the fungus uses for effector movement and hyphal invasion, thereby enabling more rapid tissue colonization by the blast fungus.

## Materials and methods

### Fungal strains, growth conditions, and infection assays

Fungal isolates were routinely grown on complete medium (CM) at 24 °C with a controlled 12 h light and dark cycle for up to 12 d (Talbot et al. 1993). *H. vulgare*, *E. curvula*, and *O. sativa* plants were grown for 7, 14, and 21 d respectively in 9 cm diameter plastic plant pots or seed trays. Conidia were recovered from 10-d-old cultures using a sterile disposable plastic spreader in 3 mL sterile distilled water. The conidial suspension was filtered through sterile Miracloth and centrifuged at 5000 × *g* for 5 min at room temperature before adjusting to a final of concentration of 1 × 10^5^ conidia mL^−1^ in 0.2% gelatin. The spore suspension was used for spray or leaf drop infections assays. After spray inoculation, plants were placed in polythene bags and incubated in a controlled plant growth chamber at 24 °C for 48 h with a 12 h light–dark cycle and 85% relative humidity, before removing polythene bags. Inoculated plants were incubated for 3 d before scoring lesions. Lesion size was estimated using hsvfinder https://github.com/danmaclean/hsvfinder. For each *O. sativa* infection, 5 to 10 leaves were collected before counting typical ellipsoid necrotic disease lesions with a gray center (Valent et al. 1991). Each experiment was repeated a minimum of 3 times, yielding consistent outcomes.

### Leaf infection assay and live-cell imaging

Rice leaf sheath from 4-wk-old susceptible cultivars Moukoto or CO39 were inoculated with 4 mL of a suspension at 5 × 10^4^ conidia mL^−1^ in ddH_2_O using a micropipette (Kankanala et al. 2007). Inoculated leaf sheaths were incubated at 24 °C for 24 h before a thin layer of inner leaf sheath was dissected and mounted on a glass slide. Treatment with INA-PP1 was carried as described previously (Sakulkoo et al. 2018). Live-cell imaging was carried out on an IX81 motorized inverted microscope (Olympus, Hamburg, Germany) for conventional and differential interference contrast (DIC) microscopy using Photometrics CoolSNAP HQ2 camera (Roper Scientific, Planegg, Germany). Images were analyzed using ImageJ. For Leica SP8 laser confocal microscopy, settings were as follows: aniline blue, GFP, YFP, and RFP/mCherry tagged proteins were excited using 405, 488, 514, and 561 nm laser diodes and emitted fluorescence detected using 440 to 490, 495 to 550, 525 to 565, and 590 to 620 nm, respectively. Auto-fluorescence from chlorophyll was detected at 650 to 740 nm.

### Generation of fungal transformation plasmids

Single or multiple DNA fragments were cloned into fungal transformation vectors using In-Fusion HD Cloning (Clontech, USA). Briefly, fragments from cDNA, genomic DNA, or synthesized DNA were amplified using primers to introduce a 15-bp overhang complementary to sequences at restriction sites of a destination vector or adjacent insert fragments. This allows the ends to fuse by homologous recombination. Positive transformants were selected by colony PCR, and constructs were sequenced by GENEWIZ. A list of primers, constructs, and restriction enzymes is provided in Supplementary Data Set 5.

### RNA isolation, RNA sequencing, and analysis

To study *in-planta* gene expression of *PWL2* and other effectors, leaf drop infection assays were carried out using susceptible rice cv. Moukoto and samples collected at 24 and 72 hpi. Infected plant material was ground to a fine powder using a sterile nuclease-free mortar and pestle containing liquid N_2_. RNA was isolated from *M. oryzae* mycelium or inoculated rice leaves using QIAGEN RNeasy Plant Mini Kit. RNA quality was determined NanoDrop spectrophotometry (Thermo Fisher Scientific, UK) and Agilent 2,100 Bioanalyser (Agilent Technologies, UK). Library preparation was carried out using Illumina sequencing TruSeq Stranded Total RNA Library Prep Kit before sequencing 100 bp paired ends reads using Illumina Genome Analyser GXII platform by Exeter Sequencing Service (University of Exeter). To determine differential gene expression, raw reads were separated by mapping to both *M. oryzae* and *O. sativa* using kraken2. Reads specific to *M. oryzae* were used to quantify transcript abundance using Kallisto. To quantify genes missing in 70-15, separated reads were mapped to KE002. R package Sleuth was used to determine genes showing differential expression with log2fold > 1 and *P*-adjust value <0.05 defined as upregulated and a log2fold > 1 and *P*-adjust value <0.05 as downregulated. Southern blot analysis of *M. oryzae* genomic DNA was carried out as described previously (Talbot et al. 1993). cDNA was synthesized using Affinity Script QPCR cDNA Synthesis Kit following the manufacturer's instructions. The reaction comprised of 10 *µ*L First-Strand Master Mix, 3 *µ*L of oligo(dT) primer, 1 *µ*L of AffinityScript RNase Block enzyme, and 3 *µ*g of RNA. The mixed reaction was incubated at 25 °C for min, 42 °C for 15 min before 95 °C incubation for 5 min for stop cDNA synthesis reaction. PCR was set to include 1× SYBR Premix ExTaq (Tli RNase plus, RR420A, Takara), 1.25 *µ*L cDNA (final 1:5 dilution), and 0.2 *µ*m of each forward and reverse primer to a final volume of 12.5 *µ*L. Quantitative real-time PCR was carried out using CFX OPUS 96, and cycle threshold values were normalized to a house keeping gene, β-Tubulin (MGG_00604). Fold change was determined using the formula 2−^ΔΔCT^, where ΔΔCt = ([CtGOI in infected sample−CtNC in infected sample]—[CtNC in mycelia−CtNC in mycelia]), and GOI is the gene of interest and negative control (NC) is β-Tubulin.

### Generation of Cas9-sgRNA–targeted gene deletion

A sgRNA was designed for CRISPR-Cas9 genome editing using online tool E-CRISP http://www.e-crisp.org/. A 20-nucleotide sequence was selected at the *PWL2* locus (not including the PAM NGG-sequence). The sgRNA was first synthesized using the EnGen sgRNA synthesis kit New England Biolabs (NEB #E3322) before mixing with Cas9-NLS (nuclear localization signal) to form an ribonucleoproteins (RNP) complex (Foster et al. 2018). The mixture was incubated at room temperature for 10 min before fungal transformation. *M. oryzae* protoplasts were generated as described previously (Talbot et al. 1993). The RNP complex together with donor template was mixed with Guy11 protoplasts resuspended in 150 *µ*L STC to a concentration of 1 × 10^8^ mL^−1^ incubated at room temperature for 25 min before adding 60% PEG. Successful transformants were selected on CM agar containing 200 *μ*g mL^−1^ hygromycin B.

### Whole-genome sequencing

Purified RNA-free DNA was obtained using hexadecyltrimethylammonium bromide. Template quality was assessed by NanoDrop and Qubit spectrophotometry. Sequencing was carried out at Exeter Sequencing services (University of Exeter, UK) and Novogene (Cambridge, UK). NEXTflex Rapid DNA-seq Library Prep Kit was used to prepare index libraries before sequencing on HiSeq 2500 (Illumina) with 2 lanes per sample. Quality of sequencing reads was checked using FastQC https://www.bioinformatics.babraham.ac.uk/projects/fastqc/. From raw data (fastq files), adaptor sequences were trimmed from sequences containing adaptors, and low-quality reads were removed by fastq-mcf. Trimmed sequences were aligned to the reference genome (70-15) (Dean et al. 2005) using Burrows Wheeler Aligner https://github.com/lh3/bwa (Li and Durbin 2009). Bam files were visualized by IGV genome viewer to determine CRISPR gene deletion.

### Copy number variation of *PWL2*

A total of 286 *M. oryzae* isolates with raw Illumina-based sequencing information were downloaded from NCBI (performed on October 16, 2019). Copy number variation was assessed using a k-mer analysis approach using the k-mer analysis toolkit (KAT; v2.4.1). Coding sequence information for *M. oryzae* isolate 70-15 was used as template for k-mer analysis and raw Illumina reads were input. Default parameters were used including k-mer length 27 nt. Copy number variation of individual effectors is based on average k-mer coverage compared with the median coverage for all genes.

### Phylogenetic analysis of *Magnaporthe* isolates

Genome sequences of diverse *M. oryzae* isolates were identified by literature review and searches on NCBI (performed on August 9, 2019). For isolates having only Illumina sequencing data, raw paired reads were downloaded from NCBI, trimmed using Trimmomatic (v0.36) using the parameters: removal of adapters with ILLUMINACLIP:TruSeq2-PE.fa:2:30:10, remove leading low quality or N based with quality below 5 for leading (LEADING:5) and trailing sequence (TRAILING:5), scan and cut reads with 4 bp sliding window below 10 (SLIDINGWINDOW:4:10), and minimum length of 36 bp (MINLEN:36). KmerGenie (v1.7048) was used to identify an optimal k-mer for genome assembly using default parameters. Genome assembly was performed using minia (v0.0.102) with default parameters. kSNP3 (v.3.021) was used to develop a phylogenetic tree of *M. oryzae* using assembled genomes as input with parameters of k-mer of 29 bp and minimum fraction of 0.4. The phylogenetic tree was generated using RAxML (v8.2.12) using the General Time Reversible model of nucleotide substitution under the Gamma model of rate heterogeneity. All sequence alignments and raw phylogenetic tree files are available (www.doi.org/10.6084/m9.figshare.28899128).

### Generation of *Agrobacterium* transformation plasmids

Single or multiple DNA fragments were cloned into binary vectors using In-Fusion HD Cloning (Clontech, USA). Briefly, fragments from cDNA were amplified to introduce a 15-bp overhang complementary to sequences at restriction sites of a destination vector or adjacent insert fragments. Alternatively, synthesized DNA fragments were designed with a 15-bp overhang complementary to sequences at restriction sites of binary vector pG514 customized for infusion cloning by digestion by either *Xba*I and *Pca*I or *Sac*I (New England Biolabs) to remove the *ccDB* toxin encoding gene. Positive transformants were accessed by colony PCR, and constructs were sequenced and analyzed on SNAP gene.

### Transient expression in *N. benthamiana*

*Agrobacterium tumefaciens* strain *GV3101* (Holsters et al. 1980) was used for transient expression. Three-week-old *N. benthamiana* leaves were infiltrated with transformed *Agrobacterium* carrying T-DNA constructs expressing the gene of interest. Bacterial cultures were diluted to obtain a final OD_600_ of 0.4 in agroinfiltration buffer (10 mm MES, 10 mm MgCl_2_, 150 *μ*m acetosyringone, pH 5.6). Leaf disks were cut from agroinfiltrated tissue 48 hpi and subjected for microscopy or used for co-IP.

### Plant transformation and ROS measurement

*A. tumefaciens* strain *AGL1* (Holsters et al. 1980) was used for plant transformation (Hensel et al. 2009). Positive transgenic plants were selected on hygromycin B followed by confirmation using PCR. Alternatively, leaf disks were collected from transgenic plants and analyzed for copy number by iDNA Genetics Ltd (Norwich, UK). In addition, expression of the protein was evaluated using SDS-PAGE. To measure the response to PTI elicitors, a 4-mm diameter biopsy punch (Integra Miltex) was used to cut leaf disks from 5-wk-old *H. vulgare* transgenic plants. Leaf disks were transferred to 96-well plates (Greiner Bio-One) containing 100 *µ*L ddH_2_O in each well and incubated overnight at room temperature. To carry out the ROS assay, ddH_2_O in wells was replaced by 100 *µ*L of 100 *µ*m Luminol or L-012 (Merck), 20 *µ*g mL^−1^ horseradish peroxidase (Merck) together with elicitors, flg22 (1 *µ*m final concentration), chitin (1 mg/mL final concentration) or without (mock). Photon count was carried out using a HRPCS218 (Photek) equipped with a 20-mm F1.8 EX DG ASPHERICAL RF WIDE LENS (SIGMA Corp.). Each experiment was repeated 3 times, yielding consistent outcomes.

### Co-IP and sample preparation for MS

Leaves were harvested from 14- to 21-d-old barley transgenic plants and rapidly frozen in liquid N_2_ before storage or immediately ground to fine powder using a GenoGrinder tissue homogenizer. Ground powder was quickly transferred to ice-cold 1.5 mL Eppendorf tubes and 2 mL of ice-cold extraction buffer (GTEN [10% {v/v} glycerol, 25 mm Tris pH 7.5, 1 mm EDTA, 150 mm NaCl], 2% [w/v] polyvinylpolypyrrolidone (PVPP), 10 mm DTT, 1× protease inhibitor cocktail [Sigma], 0.5% [v/v] IGEPAL) was added and mixed thoroughly. This was centrifuged at 3000 × *g* for 10 min at 4 °C to recover total protein in the supernatant and repeated twice. A volume of 2 mL of total protein was mixed with 25 *μ*L of GFP-TRAP agarose beads (50% slurry, ChromoTek) and incubated shaking overnight at 4 °C. The GFP-TRAP agarose beads were centrifuged for 2 min at 3000 × *g* before washing 3 times with wash buffer (ChromoTek). Bound proteins were recovered by resuspending beads in loading buffer (loading dye, 10 mm DTT, H_2_O) before incubating at 70 °C for 10 min. An aliquot was run by SDS-PAGE and used for immunoblot analysis (Burnette 1981). Recovered proteins were fractionated by SDS-PAGE for ∼1 cm. The gel was washed for 1 h in ddH_2_O followed by 1 h incubation in SimplyBlue Safe stain. The gel was then washed 3 times in ddH_2_O, and the region containing proteins excised using a scalpel.

### MS and data processing

Protein purification, immunoprecipitation, sample preparation, liquid chromatography followed by tandem MS (LC-MS/MS), and data analysis were carried out as previously described (Li et al. 2023). Briefly, proteins identified in immunoaffinity-enriched samples were measured with the data dependent method on the high resolution LC-MS systems, Orbitrap Fusion (Thermo Fisher Scientific). Acquired spectra were peak-picked and searched by Mascot (Matrix Science Ltd) to identify peptide sequences from the search space defined by background proteome. Peptides were combined into proteins based on the principle of parsimony by the search engine. Resulting proteins were further described by quantitative values based on the number of spectra that identify them. Individual runs were combined in Scaffold (Proteome Software Inc.), where the data were evaluated and filtered to contain less than 1% false positives, false discovery rate and the resulting matrix exported. The matrix of proteins detected in different samples serves as the input for an R script for further processing and visualization.

### Yeast 2-hybrid analysis

To clone genes of interest into bait (pGBKT7 DNA-BD cloning) vector or prey (pGADT7 activating domain) vector, by In-Fusion HD Cloning (Clontech), bait and prey vectors were digested by *Bam*H1 and *Eco*R1 while gene to be inserted was PCR amplified using primers containing 15 bp overhangs with homology to 2 ends of the digested bait vector. For transformation, a single colony Y2H gold yeast strain was mixed in 1 mL of liquid yeast extract peptone dextrose. Competent cells were prepared according to the manufacturer’s instructions (Zymo Research). Briefly, for transformation 700 ng to 1 *µ*g of plasmids expressing Pwl2 or other effectors in pGADT7 and *Hv*HIPP43 or plant proteins in pGBKT7 were cotransformed in competent cells, and Frozen-EZ Yeast Solution 3 was added before incubation at 28 °C for 1 h and transformed cells were plated on selection media lacking Leucine (L) and Tryptophan (W) and incubated at 28 °C for 3 to 5 d. To detect interactions, colonies were transferred to media lacking Leucine (L), Tryptophan (W), Adenine (A) Histidine (H), Bromo-4-Chloro-3-Indolyl a-D-galactopyranoside (X-a-gal), and 10 mm 3-amino-1,2,4-triazole (3AT) (Sigma). The plates were imaged after 60 to 72 incubation at 28 °C. Each experiment was conducted a minimum of 3 times.

### *In-planta* co-IP

To test interactions of proteins *in planta*, genes of interest tagged with either GFP or Myc were cloned into vector pGW514, transformed into *Agrobacterium* strain *GV3101* and coinfiltrated (OD_600_ = 0.4 for effectors and OD_600_ = 0.6 for HIPP43) into 3- to 4-wk-old *N. benthamiana* leaves and incubated for 48 h to allow protein expression. Total protein was isolated in ice-cold extraction buffer (GTEN [10% {v/v} glycerol, 25 mm Tris pH 7.5, 1 mm EDTA 150 mm NaCl], 2% [w/v] PVPP, 10 mm DTT, 1× protease inhibitor cocktail [Sigma], and 0.5% [v/v] IGEPAL). Total protein was coimmunoprecipitated using anti-GFP M2 resin (Sigma-Aldrich, St. Louis, MO, USA) and washed 3 times using immunoprecipitation buffer before analyzing by SDS-PAGE. Recovered proteins from co-IP were separated by SDS-PAGE and transferred to a PVDF membrane using a Trans-Blot turbo transfer system (Bio841 Rad, Germany). Detection was performed using the appropriate antibody of either anti-GFP-HRP or anti-Myc-HRP. Imaging was carried out using an ImageQuant LAS 4000 luminescent imager (GE Healthcare 844 Life Sciences, Piscataway, NJ, USA) according to the manufacturer's instructions.

### Subcellular localization and plasmodesmata quantification

Leaf disks were obtained from *N. benthamiana* plants agroinfiltrated with different construct combinations at 24 and 48 hpi and mounted on a slide immersed in perfluorodecalin and observed using 63× oil immersion lens. For Leica SP8 laser confocal microscopy, settings were as follows: GFP, YFP, and RFP/mCherry tagged proteins were excited using 488, 514, and 561 nm laser diodes and emitted fluorescence detected using 495 to 550, 525 to 565, and 570 to 620 nm, respectively. Auto-fluorescence from chlorophyll was detected at 650 to 740 nm. To stain and quantify PD, agroinfiltrated leaves were further infiltrated with 0.1% aniline (Sigma-Aldrich #415049, in phosphate-buffered saline buffer, pH 7). Images were analyzed using ImageJ to determine the number of PD. First, cell peripheries were divided into 20 *µ*m sections and the number of PD determined per 20 *µ*m.

### Phylogenetic analysis of grass HMA proteins

Proteins from *B. distachyon* (314; v3.1), *H. vulgare* (Barley cv. Morex V3, Jul 2020), *T. aestivum* (RefSeqv2.1; 09-16-2020), *O. sativa* (323; v7.0), *O. thomaeum* (386; v1.0), *So. bicolor* (454; v3.1.1), *S. italica* (312; v2.2), and *Z. mays* (RefGen_V4) containing an HMA domain were identified using InterProScan (v5.59-91.0; Pfam PF00403). The HMA domain was extracted using the script QKdomain_process.py (https://github.com/matthewmoscou/QKdomain) including an additional 10 amino acid sequences N- and C-terminal of the Pfam boundaries (-n 10 -c 10). The nonredundant set of HMA domains were identified using CD-HIT (v.4.8.1) with parameter -c 1.0. Structure-guided multiple sequence alignment was performed using MAFFT with parameters dash, max iteration of 1000, and global pair. Maximum likelihood phylogenetic analysis was performed using RAxML (v8.2.12) using the Gamma model of rate heterogeneity, JTT amino acid substitution model, and 1,000 bootstraps. Coding sequence was identified for the HIPP43 gene family and aligned using MUSCLE (v5) translation alignment using default parameters. Maximum likelihood phylogenetic analysis was performed using RAxML (v8.2.12) using General Time Reversible model of nucleotide substitution under the Gamma model of rate heterogeneity and 1,000 bootstraps.

### Statistical analysis and protein structure prediction

Significance difference between groups of samples was performed using GraphPad Prism 10. *P-*values of <0.05 were considered significant, ****P < 0.0001, ***P < 0.001, and **P < 0.01, while *P-*values of >0.05 were considered as nonsignificant in an unpaired Student's *t*-test. Structure prediction was carried out using AlphaFold3 (Abramson et al. 2024), the structure was analyzed, and figures were generated using ChimeraX (Meng et al. 2023). Statistical data are available in Supplementary Data Set 6.

### Accession numbers

Sequence data from this article can be found in the GenBank/EMBL data libraries under accession numbers MGG_13683/MGG_04301 (*PWL2*) and XP_044970042.1 (HIPP43).

## Supplementary Material

koaf116_Supplementary_Data

## Data Availability

All sequence alignments and raw phylogenetic tree files generated in this study are available on (www.doi.org/10.6084/m9.figshare.28899128). All other data is provided in the main article or as supplemental. Strains or plasmids generated in this study are available upon request.

## Supplementary data

The following materials are available in the online version of this article.

**Supplementary Figure S1.**  *M. oryzae* Pwl2 is coexpressed with other known effector genes during plant infection (supports Fig. 2).

**Supplementary Figure S2.**  *PWL2* is highly polymorphic compared with other members of the *PWL* family (supports Fig. 2).

**Supplementary Figure S3.** Two *PWL2* alleles are not recognized by the barley resistance gene *Mla3* (supports Fig. 2).

**Supplementary Figure S4.** Southern blot analysis to determine copy number variation of *PWL2* in selected *M. oryzae* isolates (supports Fig. 3).

**Supplementary Figure S5.**  *M. oryzae Δpwl2* deletion mutants show enhanced virulence on weeping lovegrass and cultivar Baronesse (+*Mla3*) (supports Fig. 4).

**Supplementary Figure S6.** Analysis of Pwl2 accumulation in transgenic barley plants (supports Fig. 5).

**Supplementary Figure S7.** Pwl2 interacts with 2 copies of *Hv*HIPP43 identified by IP-MS (supports Fig. 6).

**Supplementary Figure S8.** Phylogenetic analysis and expression profile of HIPP43 during rice blast infection on the susceptible rice cv. CO39 (supports Figs. 6 and 7).

**Supplementary Figure S9.** The HIPP43 isoprenylation motif is required for PD localization (supports Fig. 7).

**Supplementary Figure S10.** Pwl1, Pwl3, and Pwl4 colocalize with HIPP43 and Pwl2 colocalizes with HIPP43 orthologs from other grass species (supports Figs. 6 and 7).

**Supplementary Figure S11.** Pwl2 stabilizes and promotes HIPP43 cytoplasmic localization (supports Fig. 7).

**Supplementary Figure S12.** Pwl2 promotes HIPP43 cytoplasmic localization and mobility (supports Fig. 7).

**Supplementary Figure S13.** Pwl2 alters HIPP43 cellular localization from the PD (supports Fig. 7).

**Supplementary Data Set 1.** Blast genomes used for phylogenetic analysis (supports Fig. 2).

**Supplementary Data Set 2.** Blast genomes used for copy number variation analysis (supports Fig. 3).

**Supplementary Data Set 3.** IP-MS spectrum report (supports Fig. 6 and Supplementary Fig. S7).

**Supplementary Data Set 4.** IP-MS samples report with clusters (supports Fig. 6 and Supplementary Fig. S7).

**Supplementary Data Set 5.** Oligonucleotide primers, constructs, and restriction enzymes used in this study.

**Supplementary Data Set 6.** Statistical analysis data.

**Supplementary Video S1.** Mobility of Pwl2/HIPP43 subcellular colocalization cytosolic puncta, 48 h after coexpression in *N. benthamiana* cells and visualized using laser confocal microscopy (supports Fig. 7 and Supplementary Figs. S11 to S13).

**Supplementary Video S2.** A laser confocal microscopy time-lapse video to show lack of colocalization between AVR-PikE and HIPP43, 48 h after coexpression in *N. benthamiana* cells (supports Fig. 7 and Supplementary Figs. S11 to S13).

**Supplementary Video S3.** A laser confocal microscopy time-lapse video to show lack of colocalization between MEP3 and HIPP43, 48 h after coexpression in *N. benthamiana* cells (supports Fig. 7 and Supplementary Figs. S11 to S13).

**Supplementary Video S4.** A laser confocal microscopy time-lapse video to show lack of colocalization between a septuple mutant of Pwl2, Pwl2^SNDEYWY^, and HIPP43, 48 h after coexpression in *N. benthamiana* cells. PD are visualized as callose stained with aniline blue, Pwl2^SNDEYWY^ as green, and HIPP43 magenta fluorescence, respectively (supports Fig. 7 and Supplementary Figs. S11 to S13).

**Supplementary Video S5.** A laser confocal microscopy time-lapse video to show lack of colocalization between free-YFP and HIPP43, 48 h after coexpression in *N. benthamiana* cells. Laser confocal microscopy (supports Fig. 7 and Supplementary Figs. S11 to S13).

## Dive Curated Terms

The following phenotypic, genotypic, and functional terms are of significance to the work described in this paper:

FLS2 Gramene: AT5G46330
FLS2 Araport: AT5G46330

LAS Gramene: AT1G55580
LAS Araport: AT1G55580

BAS1 Gramene: AT2G26710
BAS1 Araport: AT2G26710

leaf AmiGo: PO:0025034

PCR Gramene: Polymerase Chain Reaction
PCR Araport: Polymerase Chain Reaction

NCBI Gramene: https://www.ncbi.nlm.nih.gov
NCBI Araport: https://www.ncbi.nlm.nih.gov

Pwl2 Gramene: GenBank: U26313.1
Pwl2 Araport: GenBank: U26313.1

MLA3 Gramene: GenBank: GU245939
MLA3 Araport: GenBank: GU245939
