# Supplementary material for: The Magnaporthe oryzae effector Pwl2 alters HIPP43 localization to suppress host immunity
Source: Plant Cell. 2025 May 9;37(6):koaf116. doi: 10.1093/plcell/koaf116 (PMC12164592; doi:10.1093/plcell/koaf116)
Supplement: koaf116_Supplementary_Data [file koaf116_supplementary_data.zip › koaf116_supplementary_data.zip!/koaf116_supplementary_data 2/TPC-2025-0030R1_Supplementary Movie Legends.pdf]

Supplementary Movie S1 (Supports Figure 7 and S11-13**)**

Mobility of Pwl2/HIPP43 sub-cellular co-localization cytosolic puncta, 48 h after co-expression in *N. benthamiana* cells and visualized using laser confocal microscopy. PD are visualized as callose stained with aniline blue, Pwl2 as green and HIPP43 magenta fluorescence respectively. Arrowheads are used to track Pwl2/HIPP43 co-localized mobile cytosolic puncta.

Supplementary Movie S2 (Supports Figure 7 and S11-13**)**

A laser confocal microscopy time-lapse video to show lack of co-localization between AVR-PikE and HIPP43, 48 h after co-expression in *N. benthamiana* cells. PD are visualized as callose stained with aniline blue, AVR-PikE as green and HIPP43 magenta fluorescence respectively. Cyan arrowheads show co-localization of aniline blue stain and HIPP43 as immobile signal at PD. Most of AVR-PikE expression occurred in the cytosol without HIPP43 co-localization.

Supplementary Movie S3 (Supports Figure 7 and S11-13**)**

A laser confocal microscopy time-lapse video to show lack of co-localization between MEP3 and HIPP43, 48 h after co-expression in *N. benthamiana* cells. PD are visualized as callose stained with aniline blue, MEP3 as green and HIPP43 magenta fluorescence respectively. Cyan arrowheads show co-localization of aniline blue stain, MEP3 and HIPP43 as immobile signal at PD. Most of MEP3 expression occurred in the cytosol without HIPP43 co-localization.

Supplementary Movie S4 (Supports Figure 7 and S11-13**)**

A laser confocal microscopy time-lapse video to show lack of co-localization between a septuple mutant of Pwl2, Pwl2^{SNDEYWY} and HIPP43, 48 h after co-expression in *N. benthamiana* cells. PD are visualized as callose stained with aniline blue, Pwl2^{SNDEYWY} as green and HIPP43 magenta fluorescence respectively. Cyan arrowheads show co-localization of aniline blue stain and HIPP43 as immobile signal at PD while expression of Pwl2^{SNDEYWY} remained in the cytosol without HIPP43 co-localization. **(**Supports Figure 7 and S11-13**)**

Supplementary Movie S5 (Supports Figure 7 and S11-13**)**

A laser confocal microscopy time-lapse video to show lack of co-localization between free-YFP and HIPP43, 48 h after co-expression in *N. benthamiana* cells. laser confocal microscopy. PD are visualized as callose stained with aniline blue, free-YFP as green and HIPP43 magenta fluorescence respectively. Cyan arrowheads show co-localization of aniline blue stain and HIPP43 as immobile signal at PD while expression of free-YFP remained in the cytosol without HIPP43 co-localization.
