# Supplementary material for: The Magnaporthe oryzae effector Pwl2 alters HIPP43 localization to suppress host immunity
Source: Plant Cell. 2025 May 9;37(6):koaf116. doi: 10.1093/plcell/koaf116 (PMC12164592; doi:10.1093/plcell/koaf116)
Supplement: koaf116_Supplementary_Data [file koaf116_supplementary_data.zip › koaf116_supplementary_data.zip!/koaf116_supplementary_data 2/Supplemental Figures.pdf]

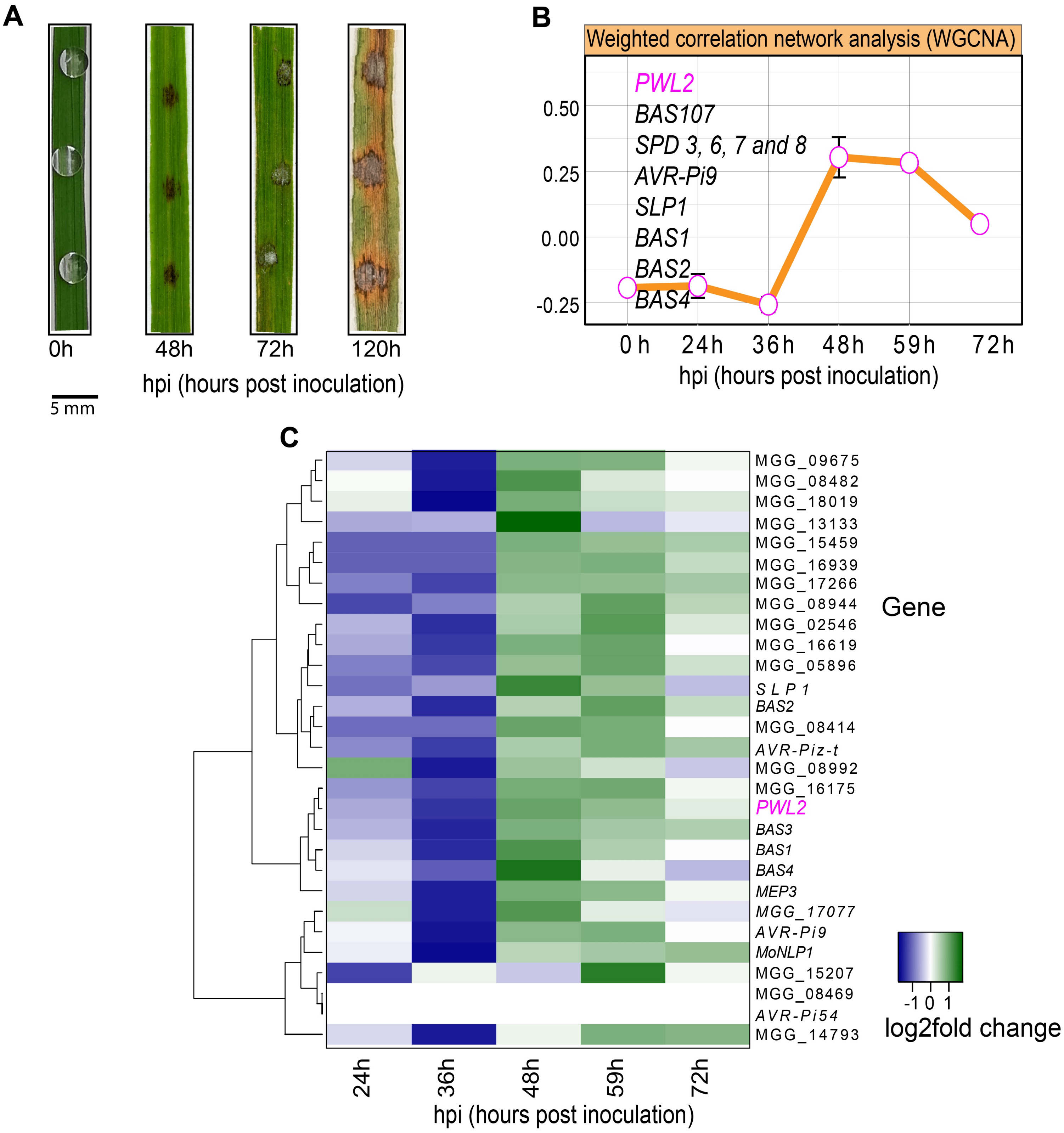
A
0h
48h
72h
120h
hpi (hours post inoculation)
5 mm
B
Weighted correlation network analysis (WGCNA)
PWL2
BAS107
SPD 3, 6, 7 and 8
AVR-Pi9
SLP1
BAS1
BAS2
BAS4
0.50
0.25
0.00
-0.25
0 h
24h
36h
48h
59h
72h
hpi (hours post inoculation)
C
MGG_09675
MGG_08482
MGG_18019
MGG_13133
MGG_15459
MGG_16939
MGG_17266
MGG_08944
MGG_02546
MGG_16619
MGG_05896
SLP1
BAS2
MGG_08414
AVR-Piz-t
MGG_08992
MGG_16175
PWL2
BAS3
BAS1
BAS4
MEP3
MGG_17077
AVR-Pi9
MoNLP1
MGG_15207
MGG_08469
AVR-Pi54
MGG_14793
Gene
-1 0 1
log2fold change
24h
36h
48h
59h
72h
hpi (hours post inoculation)

Supplementary Figure S1. ***M. oryzae*** **Pwl2 is co-expressed with other known effector genes during plant infection.** (Supports Figure 2)

(**A**) Attached leaves of three-weeks old rice seedlings inoculated with KE002 conidia suspension at 0, 48, 72 and 120 hpi (hours post inoculation). Samples were collected at 24, 36, 48, 59 and 72 hpi and used for RNA isolation (3 biological replicates) and images acquired separately at different time points. (**B**) RNA isolated from infected rice leaves were sequenced and the reads normalized against *M. oryzae* mycelia KE002 RNA to determine differentially expressed genes. A weighted gene co-expression network analysis grouped gene expression profile into 10 co-expression modules. The line graph (WGCNA) shows the representative eigengene for module 4 in which *PWL2* is co-expressed with other known effector protein encoding genes, *AVR-Pi9*, *SLP1*, *BAS1-4* and suppressors of cell death effectors 3, 6, 7 and 8 that possibly serve related function during biotrophic phase of invasion. Error bars represent SD of 3 biological replicates for all time points. (**C***)* Heatmap showing relative expression levels (log2fold change) of *PWL2* and other known effectors during infection.

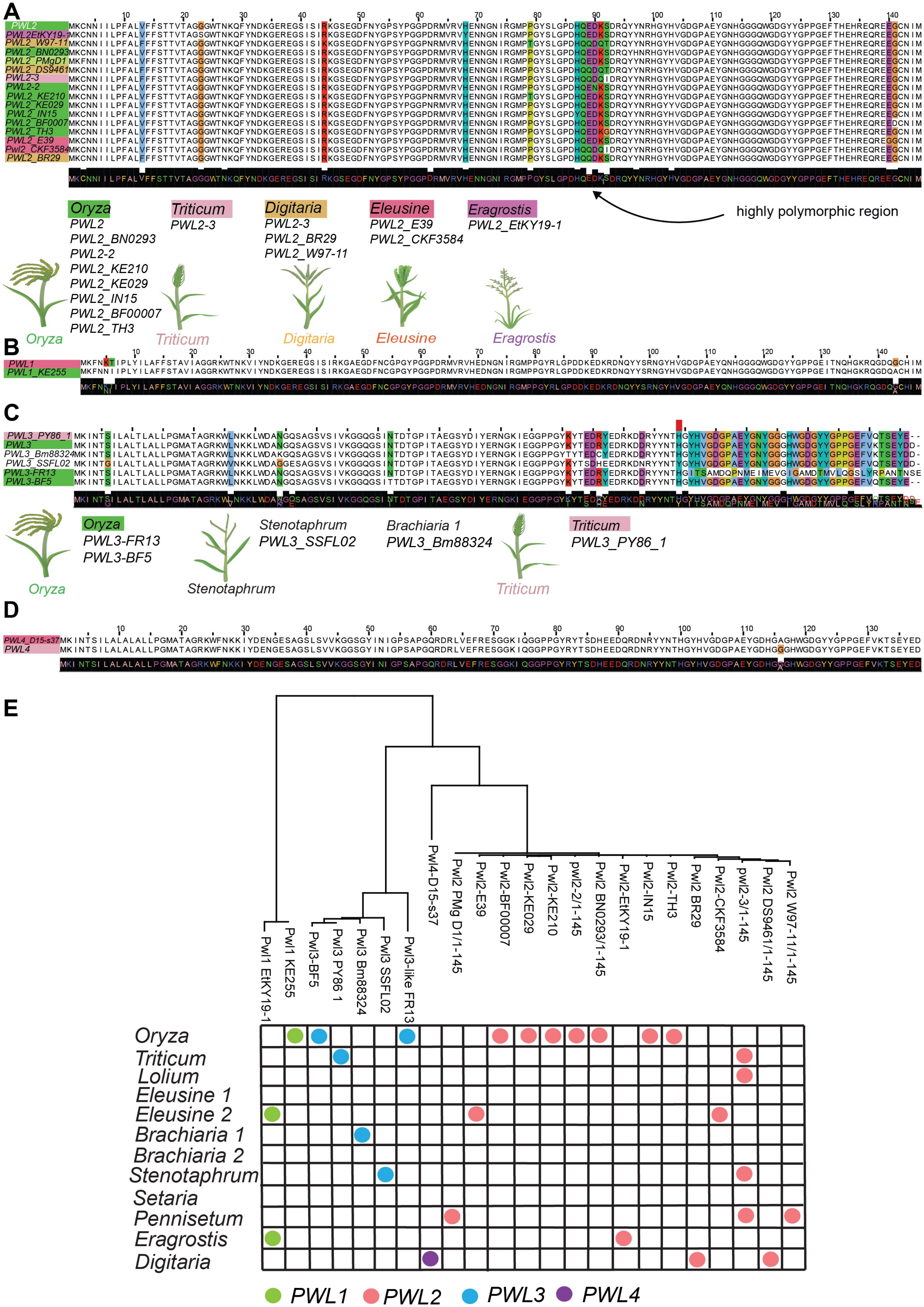
A
PWL2
PWL2EtKY19-1
PWL2_W97-11
PWL2_BN0293
PWL2_PMgD1
PWL2_DS9461
PWL2-3
PWL2-2
PWL2_KE210
PWL2_KE029
PWL2_IN15
PWL2_BF0007
PWL2_TH3
PWL2_E39
Pwl2_CKF3584
PWL2_BR29
highly polymorphic region
Oryza
PWL2
PWL2_BN0293
PWL2-2
PWL2_KE210
PWL2_KE029
PWL2_IN15
PWL2_BF00007
PWL2_TH3
Triticum
PWL2-3
Digitaria
PWL2-3
PWL2_BR29
PWL2_W97-11
Eleusine
PWL2_E39
PWL2_CKF3584
Eragrostis
PWL2_EtKY19-1
Oryza
Triticum
Digitaria
Eleusine
Eragrostis
B
PWL1
PWL1_KE255
C
PWL3_PY86_1
PWL3
PWL3_Bm88324
PWL3_SSFL02
PWL3-FR13
PWL3-BF5
Oryza
PWL3-FR13
PWL3-BF5
Stenotaphrum
PWL3_SSFL02
Brachiaria 1
PWL3_Bm88324
Triticum
PWL3_PY86_1
Oryza
Stenotaphrum
Triticum
D
PWL4_D15-s37
PWL4
E
Pwl1 EtKY19-1
Pwl1 KE255
Pwl3-BF5
Pwl3 PY86 1
Pwl3 Bm88324
Pwl3 SSFL02
Pwl3-like FR13
Pwl4-D15-s37
Pwl2 PMg D1/1-145
Pwl2-E39
Pwl2-BF00007
Pwl2-KE029
Pwl2-KE210
pwl2-2/1-145
Pwl2 BN0293/1-145
Pwl2-EtKY19-1
Pwl2-IN15
Pwl2-TH3
Pwl2 BR29
Pwl2-CKF3584
pwl2-3/1-145
Pwl2 DS9461/1-145
Pwl2 W97-11/1-145
Oryza
Triticum
Lolium
Eleusine 1
Eleusine 2
Brachiaria 1
Brachiaria 2
Stenotaphrum
Setaria
Pennisetum
Eragrostis
Digitaria
PWL1
PWL2
PWL3
PWL4

Supplementary Figure S2. *PWL2* is highly polymorphic compared to other members of the *PWL* family. (Supports Figure 2)

(**A**) Multiple amino acid sequence alignment of Pwl2 coding sequence and naturally occurring alleles. The region between His-87 and Ser-92 shows highest number of polymorphisms (shown with an arrow) and is the region important for cognate resistance gene recognition. Multiple amino acid sequence alignment of *PWL1* (**B**), *PWL3* (**C**) and *PWL4* (**D)** alleles identified from genomes of field isolates. Regions with low conservation are highlighted in colour. Alignments were carried using ClustalW (Jalview). (**E**) Neighbor-joining tree (branch lengths are not drawn to scale) based on amino acid sequence of alleles of the *PWL* gene family. Tree IDs contains isolate name from which allele was identified, and each dot represents presence in each host-limited form. Unlike other family members, *PWL2* alleles are present in host-limited forms apart from lineage 2 of *Brachiaria*, *Setaria, Panicum, Cynodon* and *Urochloa*-infecting isolates.

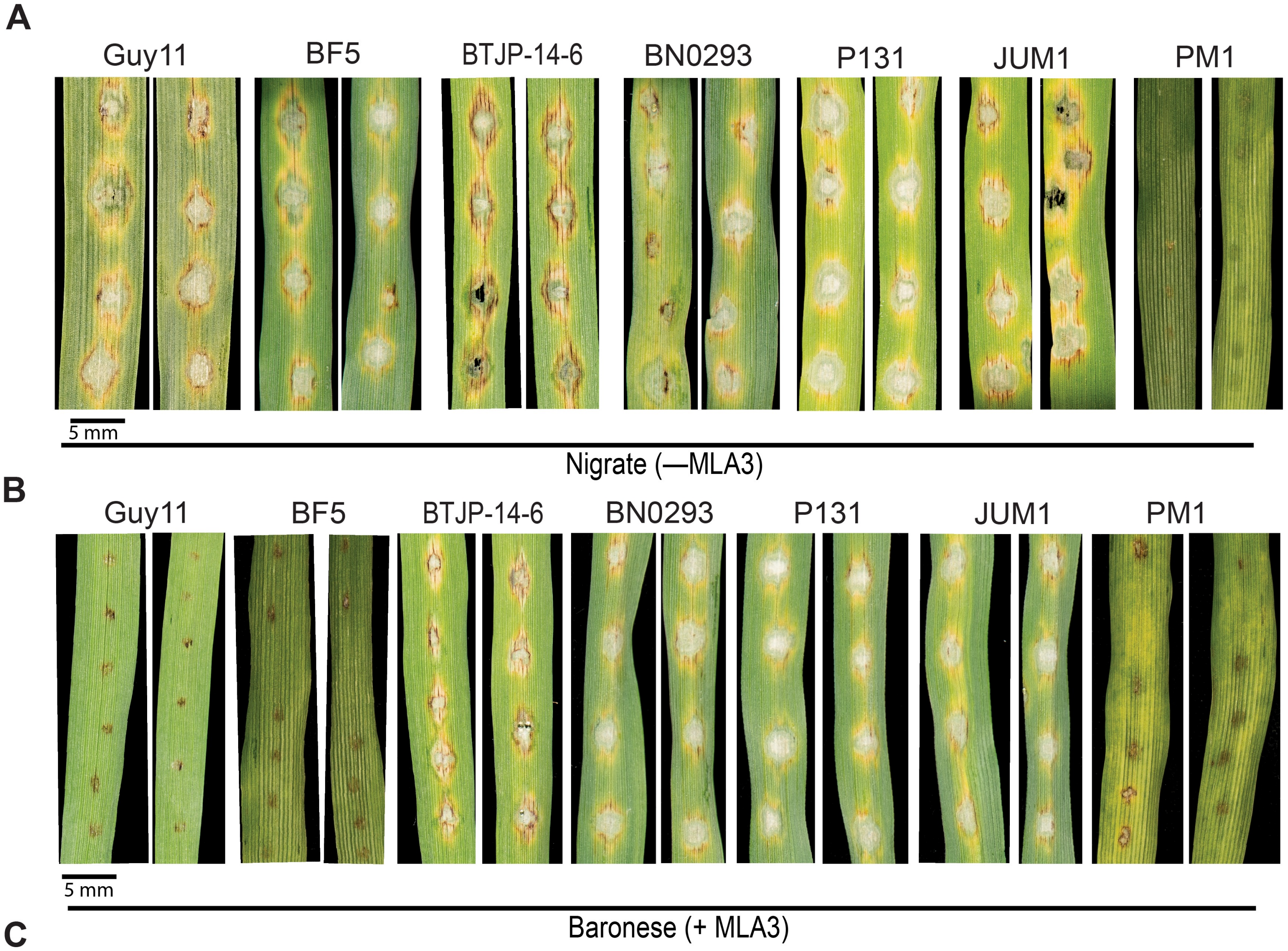

C

Isolate	Nigrate	Baronesse
Guy11	susceptible	resistant
BF5	susceptible	resistant
BTJP4-16	susceptible	susceptible
BN0293	susceptible	susceptible
P131	susceptible	susceptible
JUM1	susceptible	susceptible
PM1	resistant	resistant

Supplementary Figure S3. Two *PWL2* alleles are not recognized by the barley resistance gene *Mla3*. (Supports Figure 2)

A subset of isolates carrying alleles of *PWL2* were used in leaf drop inoculation on (**A**) barley accessions cv. Nigrate (—*Mla3*) and (**B**) cv. Baronesse (+*Mla3*). Conidial suspensions at 1 $\times 10^5$ mL^{-1} spores/mL were harvested from Guy11 and selected isolates with *PWL2* alleles and used to inoculate 10-day-old seedlings of cv. Nigrate (—*Mla3*) and cv. Baronesse (+*Mla3*). Disease lesions were scored 5 days post-infection. Isolates Guy11 (+*PWL2*), BF5 (variant *Pwl2*), BTJP4-16 (*pwl2-3)*, BN0293 (variant *Pwl2*), P131 (—*PWL2*), JUM1 (*pwl2-2*) and PM1 (variant *Pwl2*) were used for leaf drop infection assay. As expected Guy11 could infect cv. Nigrate (—*Mla3*), but not cv. Baronesse (+*Mla3*). BF5 and PM1 did not produce blast disease on barley cv. Baronesse (+*Mla3*), suggesting they express *PWL2* alleles recognised by Mla3, (or express effectors recognised by additional R-genes contained (in barley cv. Baronesse). Notably, PM1 could also not infect cv. Nigrate (—*Mla3*). P131 does not carry *PWL2* and caused blast disease on cv. Baronesse (+*Mla3*) as expected. JUM1 (*pwl2-2*), BTJP4-16 (*pwl2-3)* and BN0293 (*pwl2-BN0293*) all caused blast disease on cv. Baronesse (+*Mla3*). Scale bar represent 5 mm. (**C**) Summary of disease reaction of isolates with distinct *PWL2* alleles on Nigrate (—*Mla3*) and Baronesse (+*Mla3*). Observations were consistent in three independent infection replicates.

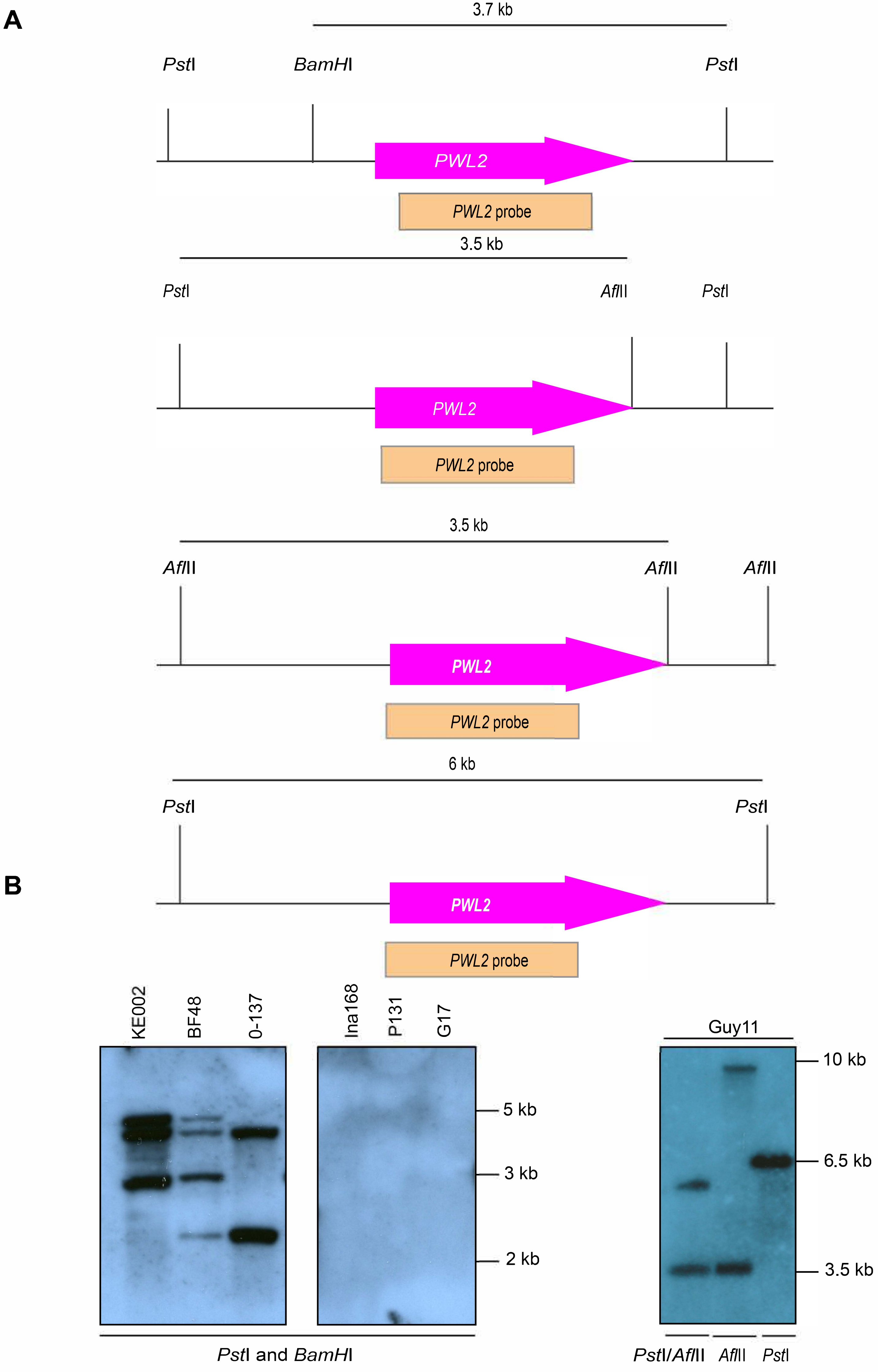
A
3.7 kb
PstI
BamHI
PstI
PWL2
PWL2 probe
3.5 kb
PstI
AflII
PstI
PWL2
PWL2 probe
3.5 kb
AflII
AflII
AflII
PWL2
PWL2 probe
6 kb
PstI
PstI
B
PWL2
PWL2 probe
KE002
BF48
0-137
Ina168
P131
G17
5 kb
3 kb
2 kb
PstI and BamHI
Guy11
10 kb
6.5 kb
3.5 kb
PstI/AflII
AflII
PstI

Supplementary Figure S4. Southern blot analysis to determine copy number variation of *PWL2* in selected *M. oryzae* isolates. (Supports Figure 3)

(**A**) Genomic DNA was digested using different enzyme combination *Bam*HI/*Pst*I (upper panel), *Pst*I/*Afl*II (upper middle panel), *Afl*II single digest (lower middle panel) or *Pst*I single digest (lower panel) and probed using the *PWL2* 438 bp probe. Size estimates are from the *PWL2* loci annotated *M. oryzae* 70-15 genome. (**B**) Left panel, the order of digested genomic DNA from different isolates is indicated on the lanes. Right panel, when Guy11 genomic DNA was digested using *Pst*1 and *Afl*II, we detected two bands of 3.5kb and 6kb. When digested with *AflII in a* single digest were detected two bands of 3.5 kb and 10 kb. While in a single digest using *Pst*I only produce a single band of 6.5 kb.

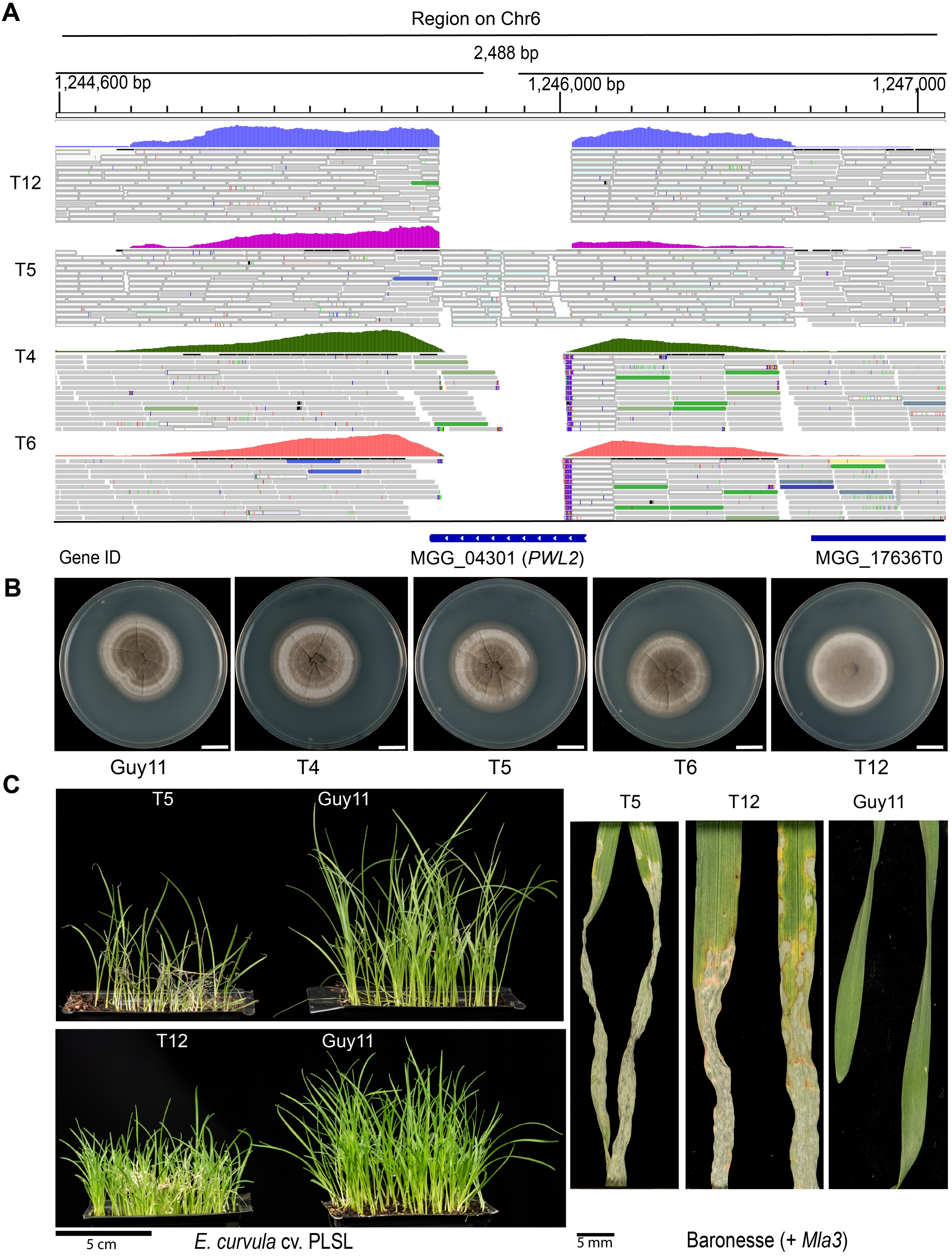

A
Region on Chr6
2,488 bp
1,244,600 bp
1,246,000 bp
1,247,000
T12
T5
T4
T6
Gene ID
MGG_04301 (PWL2)
MGG_17636T0
B
Guy11
T4
T5
T6
T12
C
T5
Guy11
T12
Guy11
5 cm
E. curvula cv. PLSL
T5
T12
Guy11
5 mm
Baronesse (+ Mla3)

Supplementary Figure S5. ***M. oryzae Δpwl2*** **deletion mutants show enhanced virulence on weeping lovegrass and cultivar Baronesse (+*Mla3*).** (Supports Figure 4)

(**A**) Reads alignment display of *PWL2* locus on Chromosome 6 from four sequenced *pwl2* mutant strains, T12, T5, T4 and T6 (lower panel). No reads aligned to the MGG_04301T0 (*PWL2*) locus for T6 and T12 and partial alignment for T4 and T5. Read depth around *PWL2* locus is higher compared to the neighbouring gene MGG_17636T0 suggesting the *PWL2* locus occurs multiple times. Read coverage and locus length are indicated. (**B**) Guy11, T4, T5, T6, T12 were grown at 25^0 C, on CM media for 10 days before imaging. Scale bars represent 1 cm. (**C**) Disease symptoms on weeping lovegrass (*E. curvula*) and barley cultivar Baronesse (+*Mla3*) to show gain of virulence for CRISPR-Cas9 deletion mutants T5 and T12. Conidial suspensions at 1×10^5 mL^{-1} spores/mL from Guy11, Δ*pwl2* (T5) and Δ*pwl2* (T12) were used to inoculate 10-day-old seedlings of barley cultivar Baronesse (+*Mla3*) or weeping lovegrass, and disease symptoms recorded after 5 dpi. Scale bars represent 5 cm or 5 mm. Observations were consistent in three independent infection replicates.

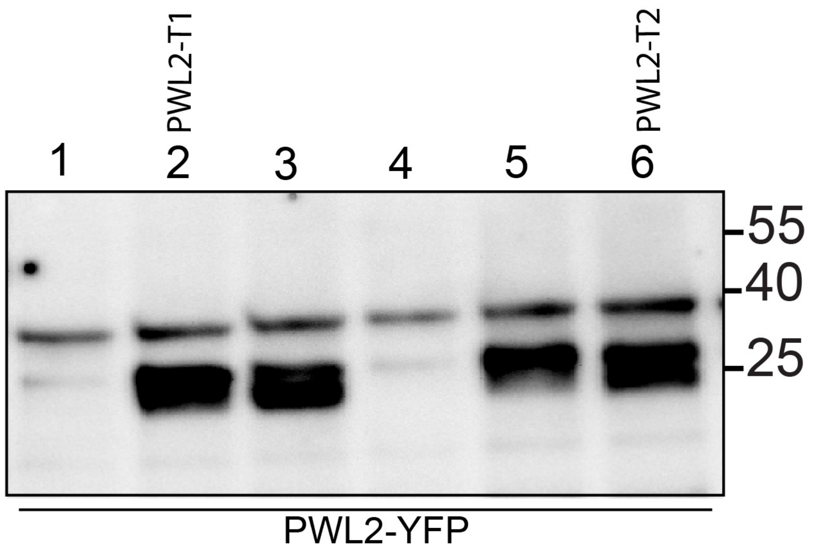
PWL2-T1
PWL2-T2
1
2
3
4
5
6
55
40
25
PWL2-YFP

Supplementary Figure S6. Analysis of Pwl2 accumulation in transgenic barley plants.

(Supports Figure 5)

Western blot to show expression of Pwl2 in transgenic barley lines. Total protein extracts from transgenic barley were immunoblotted and probed with anti-GFP-peroxidase (HRP-conjugated) antibodies. Magenta asterisks indicate expected band sizes. Lines that showed good expression were used for IP-MS experiment and test for immune response (PWL2-T1 and PWL2-T2).

A

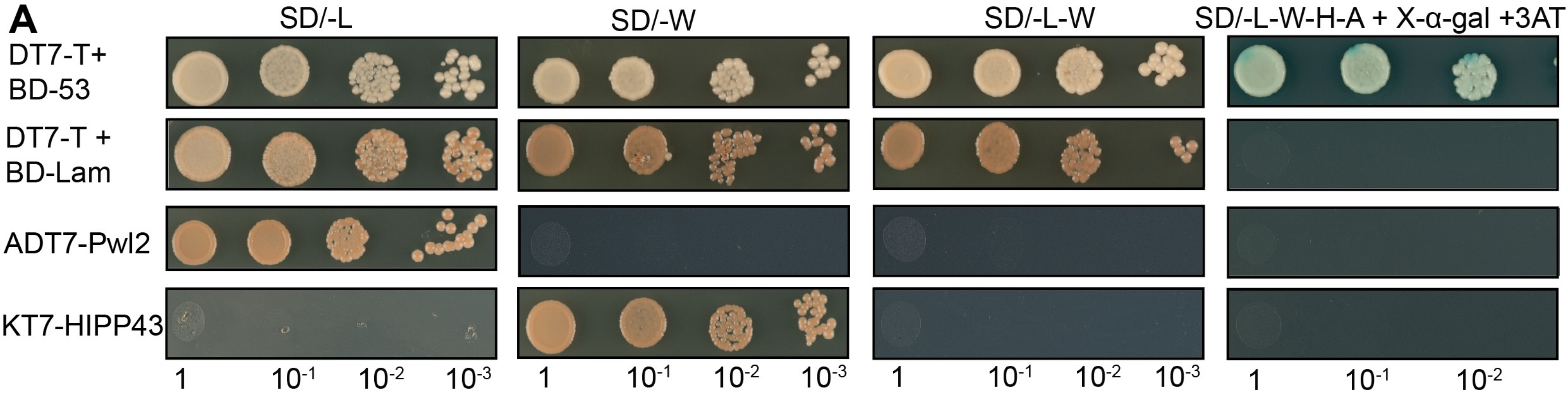

B

Accession	Description	Sequence	Peptide count
r3.2HG0164110.1 (HIPP43)	**HMA**	GAPQAmADPGAPEVR	9
		GVTAVSVNPK	2
		KAPAGFVR	2
		VTVTGFVEPSK	4
r3.3HG0267040.1	**HMA**	KAPAGFVR	2

C

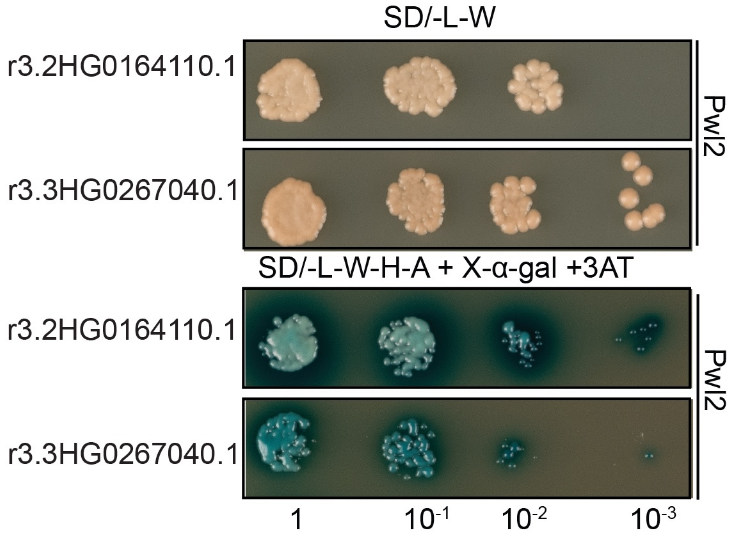

D

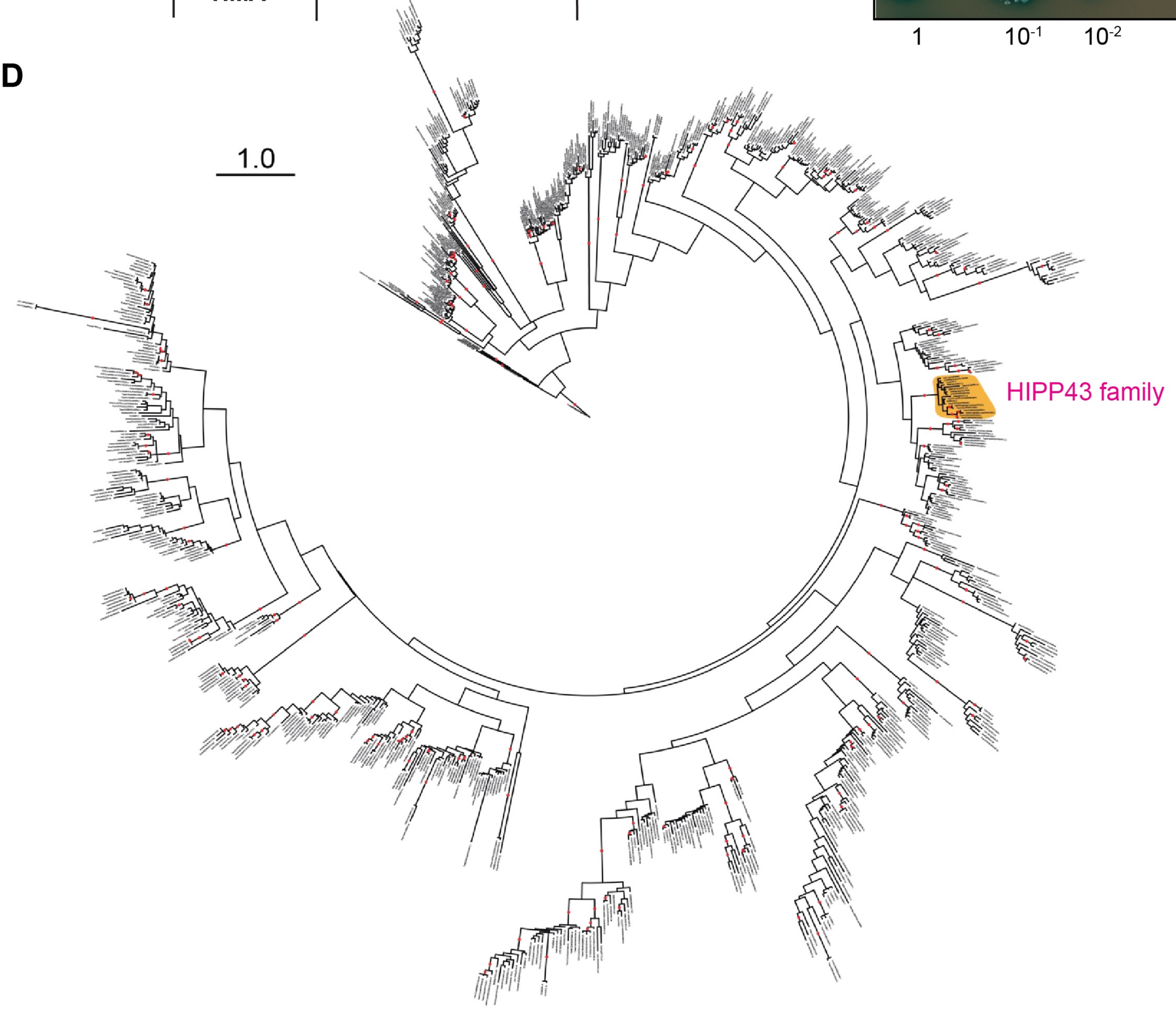

Supplementary Figure S7. Pwl2 interacts with two copies of *Hv*HIPP43 identified by IP-MS. (Supports Figure 6)

(**A**) Y2H assay to show that Pwl2 (in pGADT7 vector) and HIPP43 (in pGBKT7 vector) do not cause autoactivity (**B**) Table showing peptides identified by LC-MS that mapped to two copies of HIPP43. (**C**) Pwl2 interacts with the two copies of HIPP43 in a yeast-two-hybrid assay. (**D**) Maximum likelihood phylogenetic tree of HMA domains from diverse grass species. HMA domains were identified from proteins from the grass species *B. distachyon*, *H. vulgare*, *T. aestivum*, *O. sativa*, *Oropetium thomaeum*, *Sorghum bicolor*, *Setaria italica*, and *Zea mays*. Structure guided multiple sequence alignment was performed using MAFFT-DASH and phylogenetic analysis with RAxML (v8.2.12). Scale bar indicates substitutions per site. Dots on branches indicate support of 80% or more based on 1,000 bootstraps. The HIPP43 family formed a distinct clade with bootstrap support of 98%.

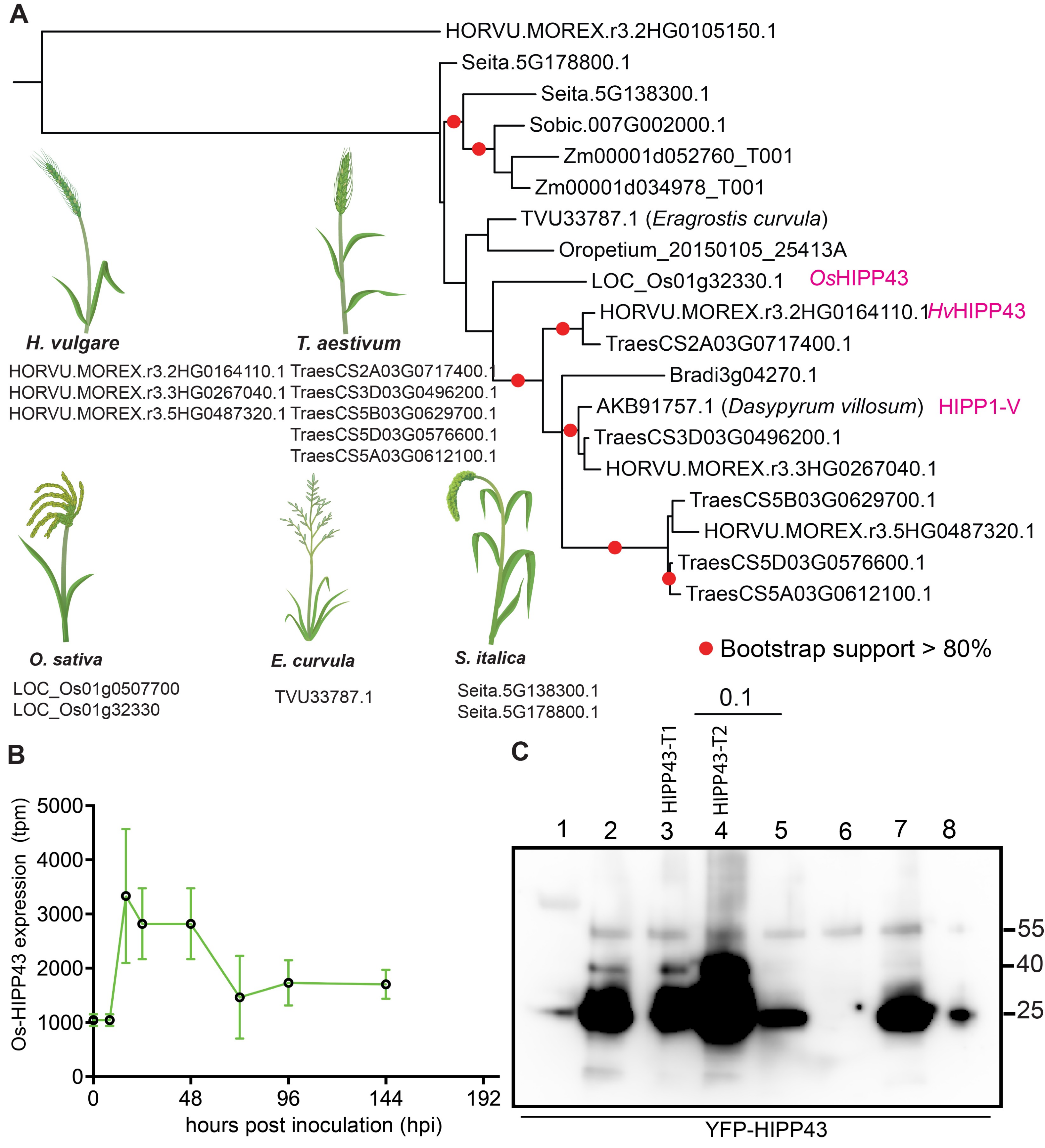

A
HORVU.MOREX.r3.2HG0105150.1
Seita.5G178800.1
Seita.5G138300.1
Sobic.007G002000.1
Zm00001d052760_T001
Zm00001d034978_T001
TVU33787.1 (Eragrostis curvula)
Oropetium_20150105_25413A
LOC_Os01g32330.1 OsHIPP43
HORVU.MOREX.r3.2HG0164110.1 HvHIPP43
TraesCS2A03G0717400.1
Bradi3g04270.1
AKB91757.1 (Dasypyrum villosum) HIPP1-V
TraesCS3D03G0496200.1
HORVU.MOREX.r3.3HG0267040.1
TraesCS5B03G0629700.1
HORVU.MOREX.r3.5HG0487320.1
TraesCS5D03G0576600.1
TraesCS5A03G0612100.1
Bootstrap support > 80%
0.1
H. vulgare
HORVU.MOREX.r3.2HG0164110.1
HORVU.MOREX.r3.3HG0267040.1
HORVU.MOREX.r3.5HG0487320.1
T. aestivum
TraesCS2A03G0717400.1
TraesCS3D03G0496200.1
TraesCS5B03G0629700.1
TraesCS5D03G0576600.1
TraesCS5A03G0612100.1
O. sativa
LOC_Os01g0507700
LOC_Os01g32330
E. curvula
TVU33787.1
S. italica
Seita.5G138300.1
Seita.5G178800.1
B
Os-HIPP43 expression (tpm)
0
1000
2000
3000
4000
5000
0
48
96
144
192
hours post inoculation (hpi)
C
HIPP43-T1
HIPP43-T2
1
2
3
4
5
6
7
8
55
40
25
YFP-HIPP43

Supplementary Figure S8 Phylogenetic analysis and expression profile of HIPP43 during rice blast infection on the susceptible rice cv. CO39. (Supports Figure 6 and 7)

(**A**) Full length coding sequence was identified for HIPP43 gene family members, aligned using MUSCLE translation alignment, and phylogenetic analysis with RAxML (v8.2.12). Barley and wheat (i.e. *Triticeae* lineage) have three copies of HIPP43 per haploid genome. Dots on branches indicate support of 80% or more based on 1,000 bootstraps and distance represents substitutions per site. (**B**) RNA-seq analysis of *OsHIPP43* expression during infection of a susceptible rice cv. CO39 by Guy11. Hour post inoculation is shown on the X –axis. HIPP43 expression is shown as relative mean expression TPM (transcripts per million) at different stages of infection. (**C**) Western blot to show expression of HIPP43 in transgenic barley lines. Total protein extracts from transgenic barley were immunoblotted and probed with anti-GFP-peroxidase (HRP-conjugated) antibodies. Magenta asterisks indicate expected band sizes. Plants that showed good expression were used to test for immune response (HIPP43-T1 and HIPP43-T2).

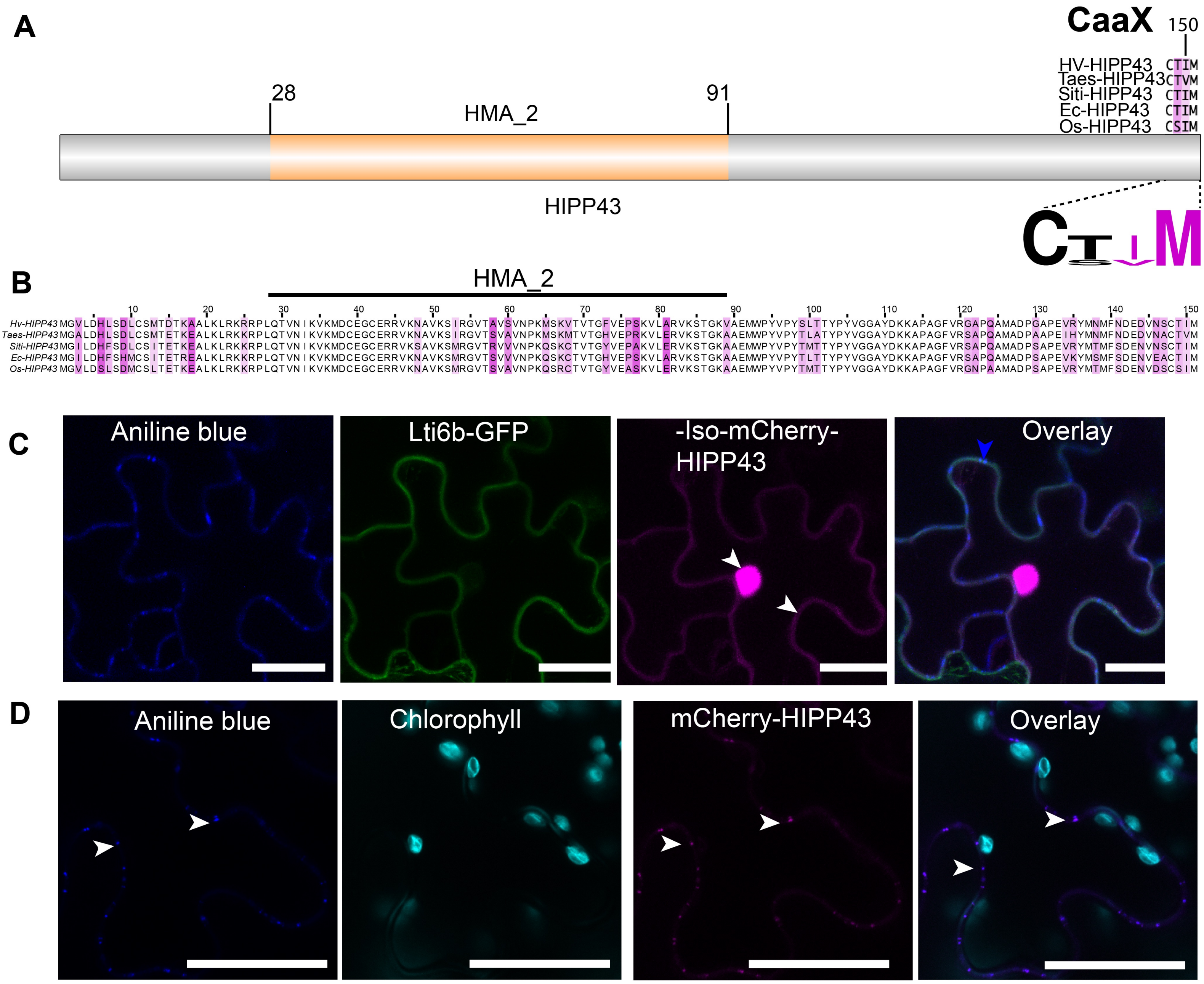
A
CaaX
150
HV-HIPP43 CTIM
Taes-HIPP43 CTVM
Siti-HIPP43 CTIM
Ec-HIPP43 CTIM
Os-HIPP43 CSIM
28
91
HMA_2
HIPP43
B
HMA_2
C
Aniline blue
Lti6b-GFP
-Iso-mCherry-HIPP43
Overlay
D
Aniline blue
Chlorophyll
mCherry-HIPP43
Overlay

Supplementary Figure S9. The HIPP43 isoprenylation motif is required for PD localization. (Supports Figure 7)

(**A +B**) A schematic representation (not drawn to scale) of domain structure of HIPP43 showing the HMA domain region (HMA_2) and the isoprenylation motif (CaaX) at the C-terminus. Isoprenylation motif of HIPP43 orthologs in rice (Os-HIPP43), wheat (Taes-HIPP43), foxtail millet (Siti-HIPP43) and weeping lovegrass (Ec-HIPP43) are indicated with varying aliphatic residue highlighted in magenta. A multiple alignment was generated using Clustal X in Jalview and the conserved motif visualized on Weblogo. Regions with low conservation are highlighted in magenta. (**C-D**) Micrographs showing localization of HIPP43 without the isoprenylation motif (-Iso-mCherry-HIPP43) and of WT mCherry-HIPP43. (**C**) Deletion of the isoprenylation abolishes PD localization but HIPP43 localizes in the nucleus and the plasma membrane after 48h. Co-expression with a plasma membrane marker LTi6b-GFP and aniline blue staining confirmed localization at the plasma membrane and lack of PD localization. White arrowheads indicate regions of HIPP43 localization away from aniline blue stained PD. **(D)** Micrographs showing typical mCherry-HIPP43 localizing as small puncta on the plasma membrane when expressed in *N. benthamiana.* Staining of callose using aniline blue overlaps with mCherry-HIPP43, confirming mCherry-HIPP43 localizes exclusively at PD. White arrowheads indicate regions of co-localization between HIPP43, and aniline blue stained PD. Scale bars represent 20 µm. Aniline blue stained PD is shown in blue, Lti6b is shown as green while mCherry-HIPP43 is shown as magenta.

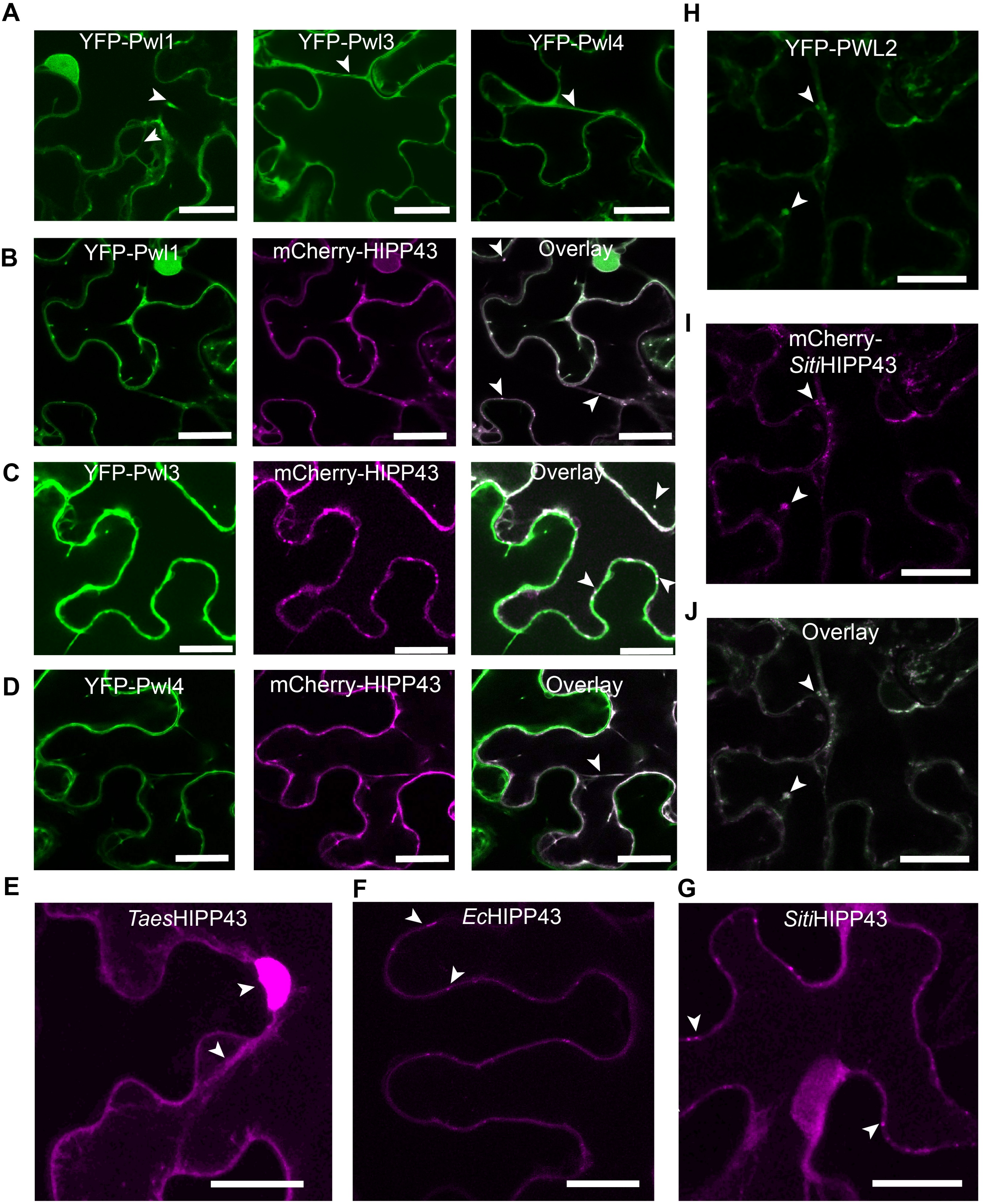
A
YFP-Pwl1
YFP-Pwl3
YFP-Pwl4
B
YFP-Pwl1
mCherry-HIPP43
Overlay
C
YFP-Pwl3
mCherry-HIPP43
Overlay
D
YFP-Pwl4
mCherry-HIPP43
Overlay
E
TaesHIPP43
F
EcHIPP43
G
SitiHIPP43
H
YFP-PWL2
I
mCherry-SitiHIPP43
J
Overlay

Supplementary Figure S10. Pwl1, Pwl3, and Pwl4 co-localize with HIPP43 and Pwl2 co-localizes with HIPP43 orthologs from other grass species. (**Supports Figure 6 and 7**)

(**A**) Micrographs showing localization of YFP-Pwl1, YFP-Pwl3 and YFP-Pwl4. Like YFP-Pwl2, Pwl1, Pwl3 and Pwl4 showed localization in cytoplasm and cytoplasmic mobile structures shown as white arrowheads **(B-D)** Micrographs showing co-localization of Pwl1, Pwl3 and Pwl4 with mCherry-HIPP43. **(B)** In presence of Pwl1 mCherry-HIPP43 translocates to the cytoplasm and partially in cytoplasmic mobile structures (shown as white arrowheads). **(C)** Pwl3 did not dislodge mCherry-HIPP43 from the PD (shown as white arrowheads), while Pwl4 **(D)** only showed cytoplasmic co-localization (shown as white arrowheads). (**E-G**) Micrographs showing HIPP43 orthologs fluorescently tagged to mCherry. (**E**) *Taes*HIPP43 (wheat) localizes in the nucleus and cytoplasm while (**F**) *Ec*HIPP43 (weeping lovegrass) (**G**) *Siti*HIPP43 (foxtail millet) localize as small puncta equivalent to PD. White arrowheads show regions of localization. Images shown as maximum projections **(H-J)** Micrographs showing co-localization of YFP-Pwl2 with mCherry-*Siti*HIPP43. mCherry-*Siti*HIPP43 localization, was altered to the cytoplasm as cytoplasmic mobile structures. mCherry-*Siti*HIPP43 fluorescence signal was higher in presence of YFP-Pwl2. White arrowheads indicate regions of co-localization and blue puncta show aniline blue stained PD. Scale bars represent 20 µm. Aniline blue stained PD is shown in blue, Pwl1, Pwl2, Pwl3 and Pwl4 are shown as green while mCherry-HIPP43 is shown as magenta.

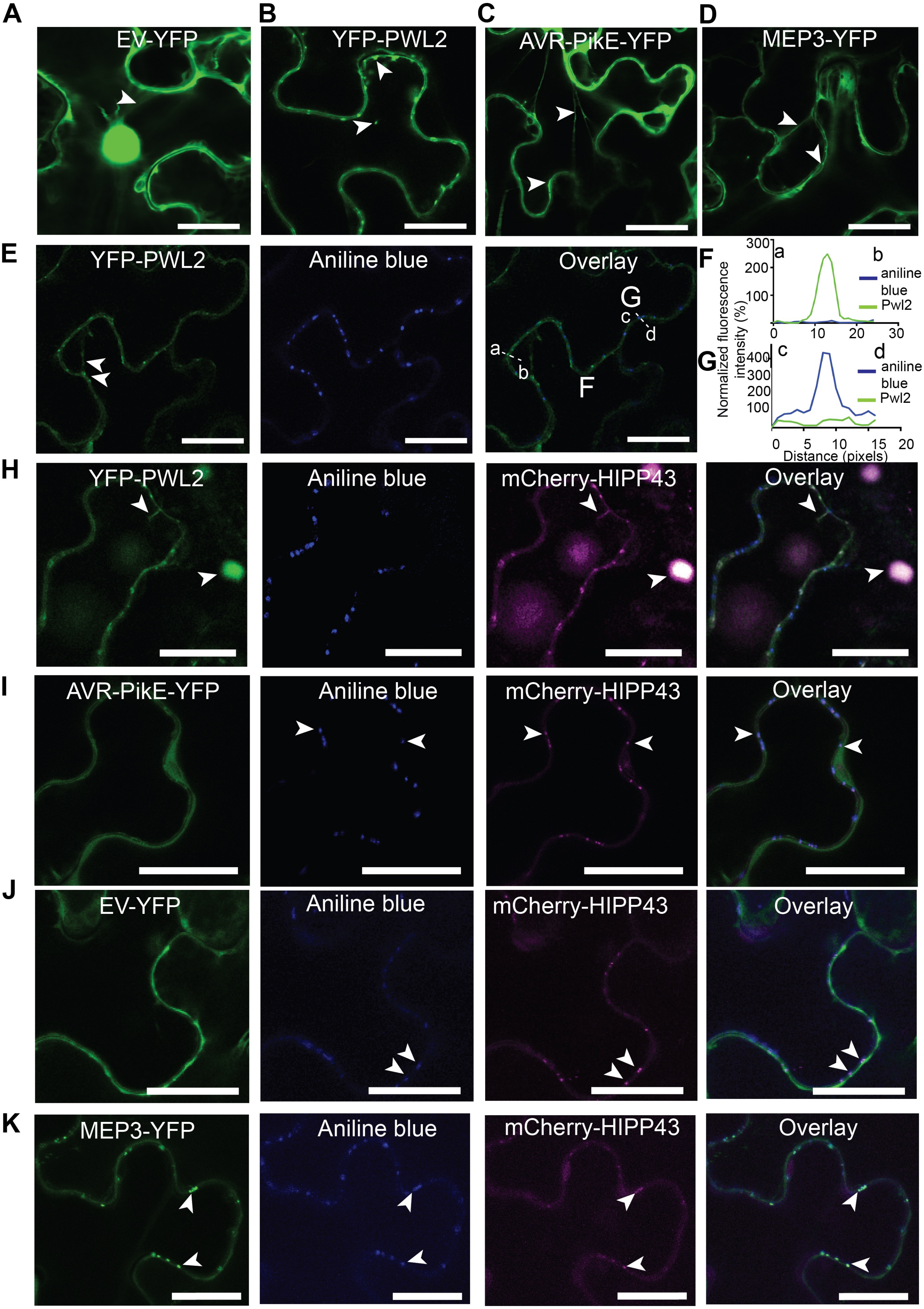

A
EV-YFP
B
YFP-PWL2
C
AVR-PikE-YFP
D
MEP3-YFP
E
YFP-PWL2
Aniline blue
Overlay
G
c
d
a
b
F
F
300
200
100
0
10
20
30
a
b
aniline blue
Pwl2
G
400
300
200
100
0
5
10
15
20
c
d
aniline blue
Pwl2
Normalized fluorescence intensity (%)
Distance (pixels)
H
YFP-PWL2
Aniline blue
mCherry-HIPP43
Overlay
I
AVR-PikE-YFP
Aniline blue
mCherry-HIPP43
Overlay
J
EV-YFP
Aniline blue
mCherry-HIPP43
Overlay
K
MEP3-YFP
Aniline blue
mCherry-HIPP43
Overlay

Supplementary Figure S11. Pwl2 stabilises and promotes HIPP43 cytoplasmic localization. (Supports Figure 7)

(**A-D)** Micrographs showing localization of free-YFP (**A**), YFP-Pwl2 (**B**), AVR-PikE-YFP (**C**) and MEP3-YFP (**D**). Both free-YFP and AVR-PikE-YFP show localization in the cytoplasm while YFP-Pwl2 showed localization in cytoplasm and cytoplasmic mobile structures (shown as white arrowheads). MEP3 localizes in the cytosol and PD (white arrowheads). **(E-G**) Micrographs and line scan graphs showing the presence of YFP-Pwl2 in the cytosol and puncta (white arrowheads). Lack of co-localization with aniline blue stained puncta (PD) confirms that, without HIPP43, Pwl2 does not accumulate at PD but rather mobile puncta. (**H-K**) Micrographs showing changes of mCherry-HIPP43 localization when co-expressed with (**H**) YFP-Pwl2, (**I**) AVR-PikE-YFP (**J**) free-YFP and (**K**) MEP3-YFP. Staining of callose using aniline blue is used to differentiate between immobile PD and mobile cytoplasmic mCherry-HIPP43 co-localization with YFP-Pwl2 (white arrowheads). In presence of AVR-PikE, free-YFP and MEP3 aniline blue staining co-localizes with immobile mCherry-HIPP43. White arrowheads indicate regions of HIPP43 localization and blue puncta show aniline blue stained PD. Scale bars represent 20 μm. Aniline blue stained PD is shown in blue, free-YFP, Pwl2, MEP3 and AVR-PikE are shown as green while mCherry-HIPP43 is shown as magenta.

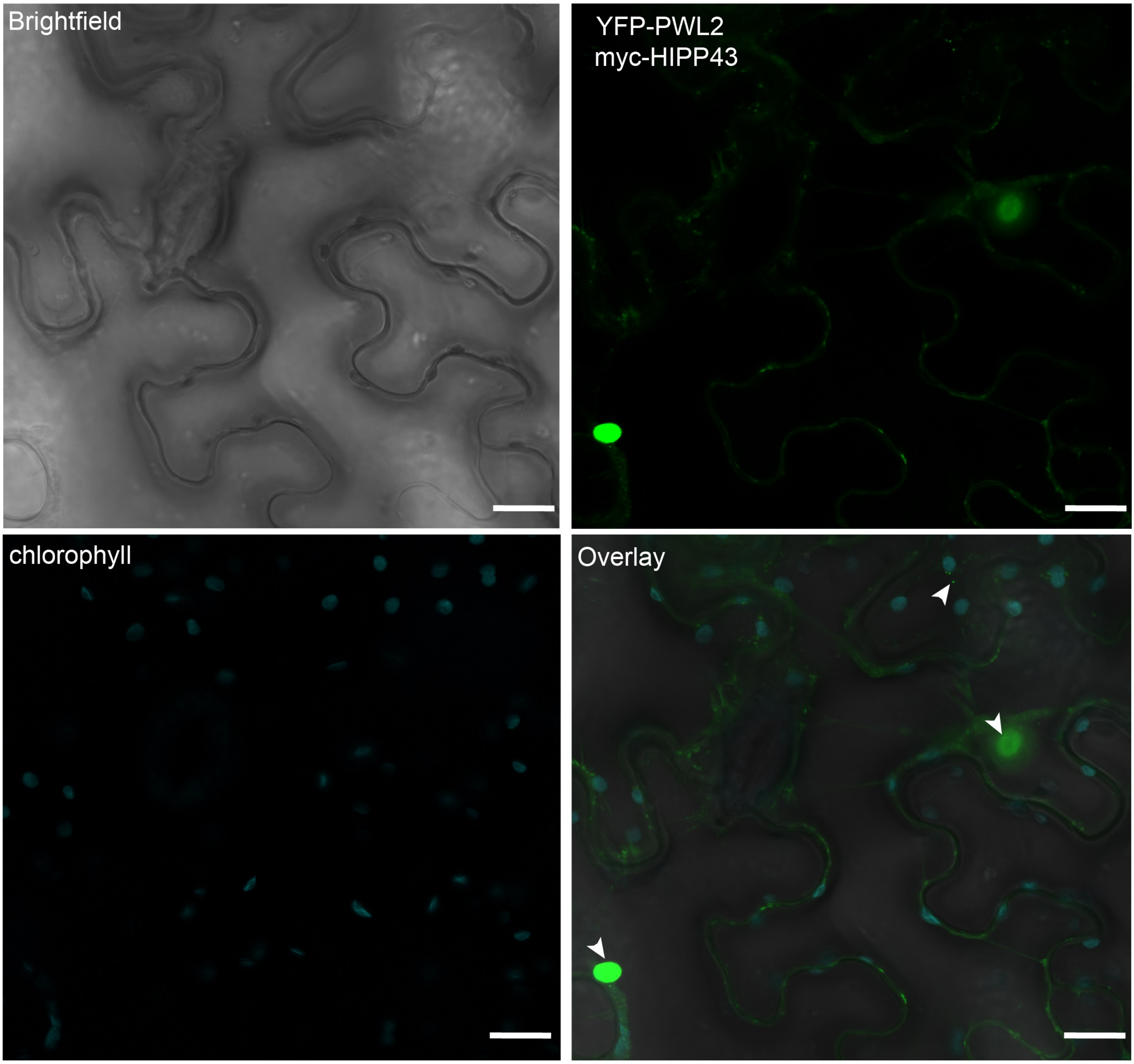
Brightfield
YFP-PWL2
myc-HIPP43
chlorophyll
Overlay

Supplementary Figure S12. Pwl2 promotes HIPP43 cytoplasmic localization and mobility. (Supports Figure 7)

Micrographs to show accumulation of YFP-PWL2 as small and big cytoplasmic mobile bodies when co-expressed with a non-fluorescent HIPP43 (myc-HIPP43). White arrowheads indicate regions with small and big cytoplasmic mobile bodies. Scale bars represent 50 µm.

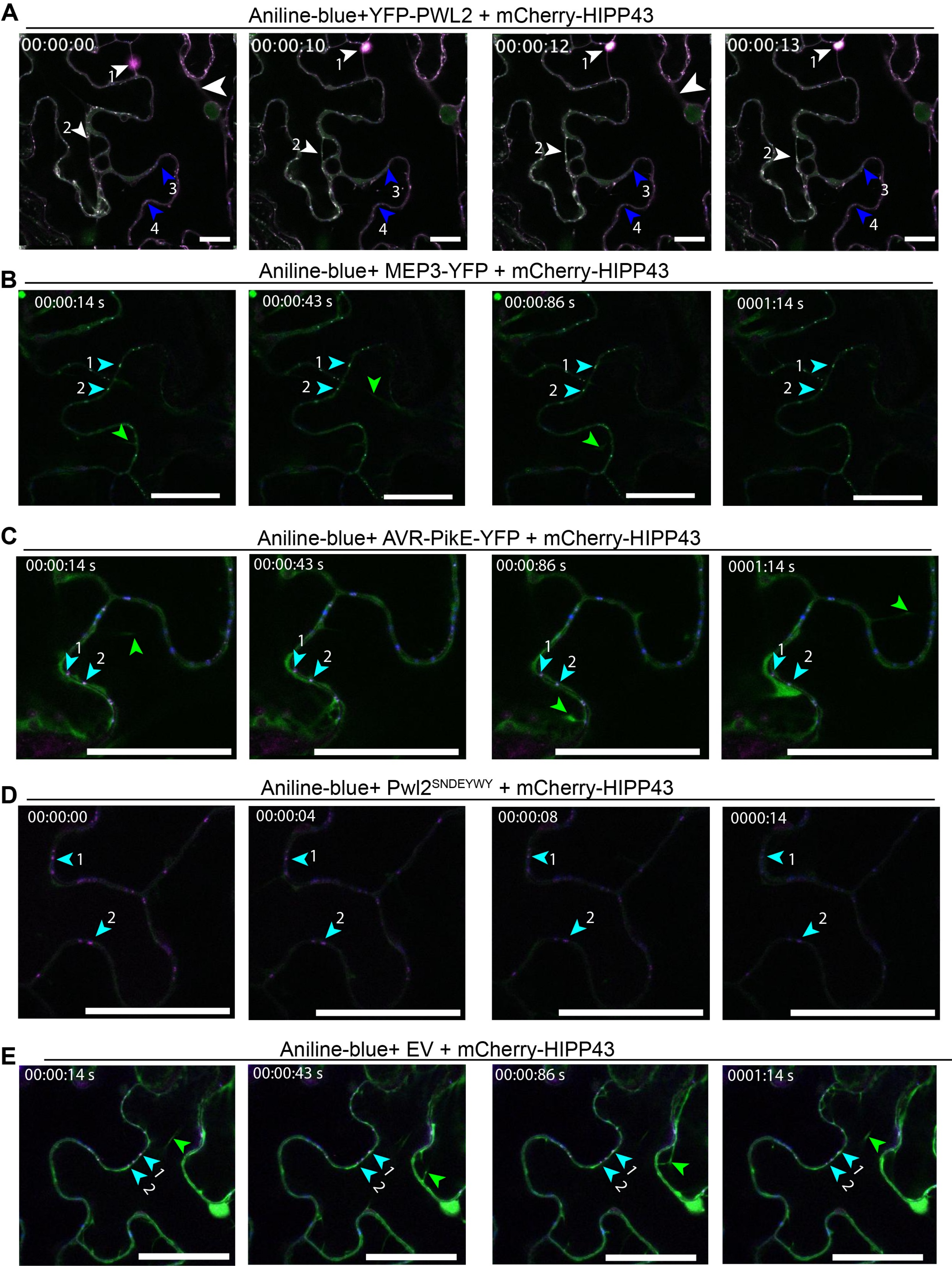
A
Aniline-blue+YFP-PWL2 + mCherry-HIPP43
00:00:00
00:00:10
00:00:12
00:00:13
B
Aniline-blue+ MEP3-YFP + mCherry-HIPP43
00:00:14 s
00:00:43 s
00:00:86 s
0001:14 s
C
Aniline-blue+ AVR-PikE-YFP + mCherry-HIPP43
00:00:14 s
00:00:43 s
00:00:86 s
0001:14 s
D
Aniline-blue+ Pwl2^{SNDEYWY} + mCherry-HIPP43
00:00:00
00:00:04
00:00:08
0000:14
E
Aniline-blue+ EV + mCherry-HIPP43
00:00:14 s
00:00:43 s
00:00:86 s
0001:14 s

Supplementary Figure S13. Pwl2 alters HIPP43 cellular localization from the plasmodesmata. (Supports Figure 7)

(**A**) A time-lapse confocal microscopy micrograph to shows changes of mCherry-HIPP43 localization upon interaction with Pwl2 after co-expression in *N. benthamiana.* Pwl2 and HIPP43 co-localize as small or large mobile puncta. White arrow heads indicate mobile Pwl2/HIPP43 co-localization puncta and blue arrowheads show immobile aniline blue stained PD. Arrowhead 1 indicate large cytoplasmic mobile body, arrowhead 2 show small mobile puncta. (**B-E**) Shows lack of co-localization between MEP3 (**B**), AVR-PikE (**C**), Pwl2^{SNDEYWY} (**D**) and (**E**) free-YFP with HIPP43. HIPP43 co-localize with PD aniline blue stain as shown with two cyan arrowheads (1 and 2) and remains immobile. Note, MEP3 localizes at PD in presence or absence of HIPP43. Green arrowheads show expression of AVR-PikE, MEP3 and free-YFP in the cytosol without HIPP43 co-localization. Scale bars represent 20 µm. Aniline blue stained PD is shown in blue, free-YFP, Pwl2, MEP3, AVR-PikE and Pwl2^{SNDEYWY} are shown as green while mCherry-HIPP43 is shown as magenta. Note that panel **E** and **Supplementary Figure S11J** are acquired from the same sample.
